# Inhibition of OGFOD1 by FG4592 confers neuroprotection by activating unfolded protein response and autophagy after ischemic stroke

**DOI:** 10.1186/s12967-024-04993-3

**Published:** 2024-03-07

**Authors:** Jian Xie, Yuan Zhang, Bin Li, Wen Xi, Yu Wang, Lu Li, Chenchen Liu, Ling Shen, Bing Han, Yan Kong, HongHong Yao, Zhijun Zhang

**Affiliations:** 1 Department of Neurology, Affiliated ZhongDa Hospital, School of Medicine, Institution of Neuropsychiatry, Key Laboratory of Developmental Genes and Human Disease, Southeast University, Nanjing, 210009 Jiangsu China; 2 Department of Pharmacology, School of Medicine, Southeast University, Nanjing, 210009 Jiangsu China; 3 Department of Biochemistry and Molecular Biology, School of Medicine, Southeast University, No. 87 Dingjiaqiao Road, Nanjing, 210009 Jiangsu China; 4 The Brain Cognition and Brain Disease Institute of Shenzhen Institute of Advanced Technology, Chinese Academy of Sciences, Shenzhen, 518055 Guangdong China

**Keywords:** FG4592, Ischemic stroke, OGFOD1, Unfolded protein response, Autophagy

## Abstract

**Background:**

Acute ischemic stroke is a common neurological disease with a significant financial burden but lacks effective drugs. Hypoxia-inducible factor (HIF) and prolyl hydroxylases (PHDs) participate in the pathophysiological process of ischemia. However, whether FG4592, the first clinically approved PHDs inhibitor, can alleviate ischemic brain injury remains unclear.

**Methods:**

The infarct volumes and behaviour tests were first analyzed in mice after ischemic stroke with systemic administration of FG4592. The knockdown of HIF-1α and pretreatments of HIF-1/2α inhibitors were then used to verify whether the neuroprotection of FG4592 is HIF-dependent. The targets predicting and molecular docking methods were applied to find other targets of FG4592. Molecular, cell biological and gene knockdown methods were finally conducted to explore the potential neuroprotective mechanisms of FG4592.

**Results:**

We found that the systemic administration of FG4592 decreased infarct volume and improved neurological defects of mice after transient or permanent ischemia. Meanwhile, FG4592 also activated autophagy and inhibited apoptosis in peri-infarct tissue of mice brains. However, in vitro and in vivo results suggested that the neuroprotection of FG4592 was not classical HIF-dependent. 2-oxoglutarate and iron-dependent oxygenase domain-containing protein 1 (OGFOD1) was found to be a novel target of FG4592 and regulated the Pro-62 hydroxylation in the small ribosomal protein s23 (Rps23) with the help of target predicting and molecular docking methods. Subsequently, the knockdown of OGFOD1 protected the cell against ischemia/reperfusion injury and activated unfolded protein response (UPR) and autophagy. Moreover, FG4592 was also found to activate UPR and autophagic flux in HIF-1α independent manner. Blocking UPR attenuated the neuroprotection, pro-autophagy effect and anti-apoptosis ability of FG4592.

**Conclusion:**

This study demonstrated that FG4592 could be a candidate drug for treating ischemic stroke. The neuroprotection of FG4592 might be mediated by inhibiting alternative target OGFOD1, which activated the UPR and autophagy and inhibited apoptosis after ischemic injury. The inhibition of OGFOD1 is a novel therapy for ischemic stroke.

**Supplementary Information:**

The online version contains supplementary material available at 10.1186/s12967-024-04993-3.

## Background

Stroke is a leading cause of death and disability worldwide, especially in China. Acute ischemic stroke accounts for the majority of strokes and shows higher morbidity and mortality [1, 2]. Interventional and thrombolysis therapies are standard for ischemic stroke patients within the time window [3]. However, few patients can benefit from reperfusion because of the narrow treatment time window or other contraindications. Meanwhile, the lack of effective neuroprotective drugs is challenging for patients and clinicians. In the last few decades, more than 1,000 effective drugs in preclinical studies failed during clinical transformations [4]. Therefore, research and development of novel neuroprotective drugs are urgently needed for patients with ischemic stroke.

Once ischemia occurs, the lack of oxygen inhibits hydroxylation of hypoxia-inducible factor (HIF) by inhibiting HIF prolyl hydroxylases (PHDs), thereby preventing the degeneration of HIF. The accumulated cytoplasmic HIF-1/2α translocates into the nucleus to combine with HIF-1β, which works as an essential transcription factor of over 100 target genes involving various pathways, including vascular endothelial growth factor (VEGF), erythropoietin (EPO), glucose transporter-1 (GLUT-1) [5]. HIF-1α has increased with monophase or biphase in the brain tissue of permanent or transient stroke models [6]. However, neuron-specific knockout of *Hif-1α* or *Hif-2α* results in unfavourable behavioral function in transient middle cerebral artery occlusion (tMCAO) mice models [6, 7]. In contrast, up-regulation of HIF-1α with the PHDs inhibitors has shown a beneficial role in infarct volume and behavioural tests in distal middle cerebral artery occlusion (dMCAO) or tMCAO mice, whether the inhibitors were administrated before or after stroke [8]. Therefore, the inhibition of PHDs may be a novel therapy for ischemic stroke.

FG4592 (also known as Roxadustat), a PHDs inhibitor, was first approved to treat anaemia caused by chronic kidney disease and showed a better effect and safety in patients [9]. Recently, basic studies have verified the protective function of FG4592 for other non-anemia diseases, including spinal cord injury, Parkinson’s disease, and retinopathy of prematurity [10]. A recent study showed that transplantation of FG4592-preconditioned bone marrow stromal cells (BMSCs) improved recovery of neurological defects in rats after ischemic stroke [11]. However, whether direct administration of FG4592 can alleviate ischemic injury after stroke remains unclear.

In the present study, FG4592 alleviated ischemic brain injury in mice subjected to permanent or transient ischemia and has a wider therapeutic time window in attenuating ischemic injury. Meanwhile, FG4592 exhibited neuroprotective function in an HIF-independent way, suggesting that some potential targets of FG4592 may play a succedaneous role. Secondly, 2-oxoglutarate and iron-dependent oxygenase domain-containing protein 1 (OGFOD1) was found to be a potential target of FG4592 via online prediction and molecular docking. Knockdown of OGFOD1 activated the unfolded protein response (UPR) and autophagy. Finally, FG4592 could activate UPR in a HIF-independent manner, further mediating the activation of autophagy flux and neuroprotection in vitro and in vivo. In summary, this study sheds light on the neuroprotective roles of FG4592 in ischemic brain injury via activating UPR and autophagy by inhibiting OGFOD1, which advances our understanding of how OGFOD1 may protect against ischemic brain injury.

## Materials and methods

### Animal preparation

According to the Stroke Therapy Academic Industry Roundtable (STAIR) recommendations, young male, female, aged mice, and mice with comorbid conditions were used in this study [12, 13]. Male C57BL/6JGpt mice (8-week-old male mice, weighing 23–25 g), old C57BL/6JGpt mice (12-month-old male mice, weighing 30–34 g), female mice (8-week-old male mice, weighing 23–25 g), High-Fat Diet-Induced Obese (DIO) male mice (14-week-old male mice fed with high-fat diet [60 kcal % Fat] for 8 weeks to induce obesity, weighing 40–43 g) and C57BL/6JGpt mouse embryos (E17-18) were obtained from the Gempharmatech Co. All experimental procedures in the present study were approved by the Institutional Animal Care and Use Committees at Southeast University and performed at a pathogen-free facility. The number of animals was determined by a power analysis based on our previous experience with variance in outcomes and effect sizes in the tMCAO or dMCAO mice model. All experiments and data analyses were performed in a blind and randomized manner.

### Establishment of transient middle cerebral artery occlusion (tMCAO)

The C57BL/6JGpt 8-week-old male mice, weighing 23–25 g, were anesthetized by the mixture of oxygen and isoflurane (2.5%) during the surgical operation. The right common carotid artery (CCA) was isolated and temporarily occluded by a 5–0 suture. The tiny branches of the external carotid artery (ECA) were mutilated with electric coagulation. The ECA was ligated with two hard knots, and the ECA between two knots was cut off. The proximal part of the right ECA was temporally occluded with the microvessel clamp. A small hole was opened on the wall of ECA between the proximal knot and clamp with scissors. A 15 mm-long 6–0 silicon-coated (about 5-6 mm is coated silicon) monofilament suture (602256PK5Re. Doccol Corporation) was inserted into the hole of ECA, and the head of the suture was turned to the internal carotid. After the opening of the clamp, the suture was inserted slowly and stopped when resistance appeared. Meanwhile, the suture left outside was about 5 mm and fixed tightly with a reversible knot. The mice's wound was sutured carefully, and mice were placed under an infrared heating lamp during the post-occlusion period (60 min). After the ischemic period, mice were anesthetized according to the previous methods, and the ECA was exposed again. The reversible knot was unclasped, and the suture was removed slightly. Meanwhile, the knot was tied off permanently and immediately to prevent bleeding, and the suture on the CCA was removed, allowing blood reperfusion. The wound was sutured carefully again, and mice were placed back in the warm cage with a soft diet. For sham operations, all procedures were identical except for the occlusion of CCA and the insertion of the monofilament suture. An experimenter blinded for the subsequent treatments randomly assigned mice with the successful operation to different groups by coin flipping or roll of a dice.

### Measurement of CBF

According to a previous study, the mouse brain's cerebral blood flow (CBF) was measured at preoperative, intraoperative and postoperative stages [14]. Briefly, the mouse was anaesthetized by the mixture of oxygen and isoflurane (2.5%) and whose hair was removed. The mouse's scalp was sterilized and cut along with the sagittal line of the head. The head of the mouse was placed under a charge-coupled device camera of Laser Speckle Contrast Imaging/LSCI (RFSLI ZW/RFLSI III, RWD Life Science CO., LTD, China). The intact skull was exposed under the laser diode (785 nm), which can penetrate through the whole brain. The CBF was measured 15 min before MCAO, 15 min after MCAO and 15 min after the reperfusion. The CBF was evaluated in the region of interest, which is posterior to the coronal suture and medial to the linear temporalis. Mice that did not show a CBF reduction of at least 75% of the baseline level or died after ischemic induction (< 10%) were excluded from further experimentation because the probability of infarction is greater than 95% if early CBF falls below 25% of the baseline.

### Establishment of distal middle cerebral artery occlusion (dMCAO)

Current STAIR recommends that any treatments of ischemic stroke should be verified in both permanent and transient occlusion (reperfusion) models. Therefore, the permanent occlusion of MCA was achieved by electrocoagulation [12]. Briefly, the hair on the right head of the mouse was shaved between the eye and ear after the mouse was anaesthetized. An incision was made from the corner of the eye to the anterior of the ear horizontally, and the incision was dragged with two drag hooks. The squamosal bone was exposed after removing the temporal muscle. The squamosal bone near the middle cerebral artery (MCA) was thinned using an electronic-powered dental drill. A small burr hole was made approximately 3 mm in diameter after removing thin bone with tweezers. The overlying dura mater was removed to expose the MCA. The MCA was occluded above and below the rhinal fissure with an electronic coagulator. After confirmation of no recanalization in MCA, the skin incision was closed with 3–0 sutures. Sham-operated mice received the same surgical procedure besides arterial occlusion. All animals were subsequently placed back in the warm cage and supplied with water and food. An experimenter blinded for the subsequent treatments randomly assigned mice with the successful operation to different groups by coin flipping or roll of a dice.

### Drugs

Roxadustat (FG4592), Lificiguat (YC-1), PT2385 and 4-phenylbutyric acid (4-PBA) were purchased from the Selleck Company (Selleck chemicals IIc, Houston, TX, USA).

### Experimental design and drug administration

FG4592 was injected into the tail veins of mice after 5 min of the tMCAO or dMCAO. According to the clinical dose of patients (BW = 60 kg, 100 mg, po, once every 2 days), a mouse was given about 10 mg/kg FG4592 based on the body surface area. Moreover, a dose gradient of FG4592 (2.5, 5, 10 mg/kg) was used in the tMCAO mice. FG4592 was dissolved in dimethyl sulfoxide (DMSO, final concentration of DMSO = 1%) and then diluted in sterile 1 × PBS (PH = 7.45) before the injection. The final solution of FG4592 and vehicle (1% DMSO in 1 × PBS) were injected into the tail vein based on the body weight of the mice. Meanwhile, edaravone was dissolved in DMSO and then diluted in 1 × PBS (final concentration of DMSO = 1%). Edaravone was used as a positive control drug and injected into the tail vein at 3 mg/kg body weight twice (5 min and 30 min after the reperfusion) [15]. After the surgery and treatment of drugs, the infarct volume and neurological defects were assessed the next day. Moreover, mice were treated with FG4592 (5 mg/kg) at 0 h, 3 h, 6 h and 12 h after reperfusion of tMCAO to ensure the therapeutic time window. The injection volumes of vehicle, FG4592 and edaravone were equivalent. FG4592 was dissolved in DMSO and DMEM (the final concentration of DMSO = 1 ‰) for the in vitro studies.

To evaluate the long-term neuroprotection of FG4592 in mice following tMCAO or dMCAO. FG4592 (5 mg/kg, *i.v.*) and vehicle (1% DMSO in 1 × PBS, *i.v.*) were administrated once every 2 days for 28 days, and edaravone (6 mg/kg, *i.v.*) was injected from 1 to 14 days after reperfusion once daily. The injection volumes of vehicle, FG4592 and edaravone were equivalent.

To examine whether the neuroprotective function of FG4592 is HIF-1α dependent or not, YC-1 (Lificiguat) was used to down-regulate the expression of HIF-1α [16]. According to the previous study, YC-1 (1% DMSO in 1 × PBS. 10 mg/kg, *i.p.*) was administered 1 h before the surgery. The injection volumes of vehicle, FG4592 and YC-1 were equivalent. YC-1 was dissolved in DMSO and DMEM (the final concentration of DMSO = 1 ‰) for the in vitro studies.

4-PBA is a small molecular inhibitor of UPR [17, 18]. 4-PBA was administrated (dissolved in 1% DMSO and then diluted in 1 × PBS. *i.p.*) 1 h before the ischemia. The injection volumes of vehicle, FG4592 and 4-PBA were equivalent. For in vitro experiments, 4-PBA was dissolved in DMSO and DMEM (the final concentration of DMSO = 1 ‰).

### Infarct volume measurement

After 24 h of reperfusion or dMCAO, mice were anesthetized with pentobarbital sodium, and the brains were rapidly removed to stain. Briefly, brains were cut into 1 mm sections coronally with the blade. Brain sections were then incubated in 1% 2,3,5-triphenyl Tetrazolium chloride (TTC; Sigma-Aldrich, T8877, dissolved in 1 × PBS) and placed in a 37 ℃ incubator. All slices were turned over every 2 min for complete staining during this procedure. The sections were scanned digitally after fixing in 4% formaldehyde (PFA; Sigma-Aldrich,158127) overnight. The sections were scanned digitally on the second day. The digital pictures were analyzed using the ImageJ software (National Institutes of Health) by an observer blinded to the groups of all animals. The infarct volume was calculated by subtracting the non-infarcted area in the ipsilateral hemisphere from the total area of the contralateral hemisphere.

### Atrophy volume measurement

After the long-term behaviour tests, the mouse brain was perfused with 1 × PBS and fixed by 4% PFA overnight. All brains were dehydrated with 30% sucrose (BioFoxx, 1245GR500) and removed to -80 ℃ overnight. A series of 30 μm thick coronal brain slices were collected for a total thickness of nearly 6.0 mm. No.1, 11, 21…, 191 brain slices were collected and stained with the cresyl violet staining solution (Beyotime, C0121). All slices were photoed with the bright field of the microscope (OLYMPUS, Tokyo, Japan, DP73) and calculated using ImageJ software by an experimenter blinded for the groups. The calculation of brain atrophy volume was performed by using the following formula: V = ∑ 0.03 × 10 [(ΔS_n_ + (ΔS_n_ + ΔS_n+1_) ^1/2 + ΔS_n+1_], where ΔS represents the contralateral area minus the atrophic area of every slice [19].

### Neurological behavioral test

All mice were trained for 3 days according to the requirements before the surgery. For evaluation of long-term neurological function, behavioral tests were performed on days 3, 7, 14, 21, and 28 after stroke onset by another two investigators blind to the experimental groups using the modified neurological severity score (mNSS), cylinder test, adhesive removal test and foot fault test.

### mNSS

The mNSS was used to grade neurological deficits, including motor (flexion of limbs and head, walking) sensory tests (performance on a balance beam). The minimal score is 0, and the maximal core is 12. The aggregate score reflects combined motor and sensory function.

### Cylinder test

To assess the symmetry of the forelimb. Mice were placed in the transparent cylinder, and the frequency of the ipsilateral, contralateral or bilateral forelimbs to explore was noted until the sum of touch was 20 times. The asymmetry score was calculated by the formula: ((the number of contacts with the contralateral limb)—(the number of contacts with the ipsilateral limb)/(the number of total contacts)) [20].

### Adhesive removal test

The adhesive removal test was employed to assess somatosensory deficits and motor coordination [21]. Mice were placed in a transparent box (30 × 20 × 25 cm) after a habituation period of 60 s. Two adhesive and colourful round papers (diameter = 0.6 cm) were randomly pasted with equal pressure on each forepaw. After that, the mice were placed back in the box, and the time that the mice spent removing the paper was recorded (maximum: 300 s). The difference between the two limbs was calculated as follows: D-Value = (Time of left limb- Time of right limb).

### Foot fault test

The foot-fault test was used to evaluate the accuracy of the forepaw. Briefly, mice were placed on a wire netting where the grid size is 1 × 1 cm and walked freely. The left forepaw’s foot faults and normal steps were counted until the total steps were approximately 150. The percentage of foot faults of the left forepaw to total steps was used as the final index [22]. A ratio between foot faults and total steps was calculated as follows: the number of foot faults/the total number of steps.

### Cell culture

HT-22 (Shanghai Jinyuan Biotechnology Co., LTD. JY111) cells were maintained in DMEM basic (Gibco, C11995500BT) supplemented with 10% FBS (Gibco, 10,099–141) and 50 mg/ml penicillin/streptomycin (NCM Biotech, C125C5) at 37 ℃ in 5% CO_2_.

### Primary neuron cultures

Primary cortical neurons were harvested from embryonic day 17–18 mouse pups. Cerebral cortices were collected and cut into small pieces that were dissociated by 0.05% trypsase (Gibco, 25200–072) with the pipette. After termination with FBS, cells were centrifuged at 1000 rpm for 5 min and plated with DMEM media (Gibco, 11965092) on dishes pre-coated with poly-D-lysine (100 mg/ml, Gibco, A3890401). After incubation at 37 ºC for at least 6 h, the plating medium was changed to Neurobasal (Gibco, 21103-049) containing 2% B27 (Gibco, 12587001), 25 mg/ml penicillin/streptomycin and 0.25% glutamine (Gibco, A29168-01). Half the volume of the culture medium was changed every 3 days.

### Oxygen–glucose deprivation/reoxygenation

Oxygen–glucose deprivation/reoxygenation (OGD/R) was applied to the primary cortical neuron or HT-22 cell. In brief, the culture medium was removed, and dishes were washed with 1 × PBS three times. The glucose-free DMEM (Gibco,11,966,025) was added to the dishes. The culture was then incubated in an oxygen-deprived box (Billups-Rothenberg, 92,014) filled with 5% CO_2_ and 95% N_2_ at 37 ℃ for 3 h. All medium was replaced with Neurobasal or DMEM medium and then incubated at 37 ℃ with 5% CO_2_ for 6 h. All drugs were added into the culture medium 1 h before OGD/R for the pre-treatment. The post treatments of drugs were carried out immediately after the change to glucose-free DMEM.

### Transfection of siRNA and mRFP-GFP-MAP1LC3B adenovirus

A mixture of Lipofectamine 2000 transfection reagent (Thermo Invitrogen, 11668019) and siRNA (GenePharma, Suzhou, Jiangsu, China) was added to HT-22 cells in DMEM without FBS. After 6 h of culture, the medium was changed to DMEM containing 10% FBS, and the cells were incubated at 37 ℃ in a 5% CO_2_ incubator. The RNA and protein samples were collected and used for further experiments after 36 or 48 h of transfection. Specific siRNA sequence targeting PHD2 was designed as follows. Hif-1α-mus, sense (5'-3'): GCCGCUCAAUUUAUGAAUATT, antisense (5'-3'): UAUUCAUAAAUUGAGCGGCTT. Phd2-mus-788, sense (5'-3'): CUGGGCAACUACAGGAUAATT, antisense (5'-3'): UUAUCCUGUAGUUGCCCAGTT. Phd2-mus-1070, sense (5'-3'): GCCACAAGGUACGCAAUAATT, antisense (5'-3'): UUAUUGC -GUACCUUGUGGCTT. Phd2-mus-1174, sense (5'-3'): GCCCAAUUCAGUCAGCAAATT, antisense (5'-3'): UUUGCUGACUGAAUUGGGCTT. Ogfod1-mus, sense (5'-3'): CCACUGAUAUCACUGAAGAUU, antisense (5'-3'): UCUUCAGUGAUAUCAGUGGUU.

For evaluation of the autophagic flux dynamically. The tandem fluorescent-mRFP- GFP-MAP1LC3B adenovirus (HanBio, HB-AP2100001) was used to transfect the primary neurons cultured on the glass coverslips within the 24-well plate for 14 days. The adenovirus (MOI = 100, within 250 μl neurobasal) was added to each well, which had been refilled with a new Neurobasal. After 10 h, the culture medium was changed with a new Neurobasal containing B27, penicillin/streptomycin and glutamine. The coverslips were used for the subsequent experiments after 4 days of transfection. The mRFP positive (red) or mRFP-GFP double positive (yellow) dots represent autolysosomes and autophagosomes, respectively. The numbers of both dots were counted to reflect the intensity of autophagic flux [23].

### Cell viability assay

Cell viability was measured with Enhanced Cell Counting Kit-8 (Beyotime Biotechnology, C0042, Shanghai, China). Briefly, 5 × 10^3^ HT-22 cells were planted in each well of the 96-well plate. The 10 μl CCK-8 solution was added to every well after the OGD/R and treatments according to the manufacturer’s instructions. The absorbance was read at 450 nm by the microplate reader (BioTek, America).

### Lactate dehydrogenase (LDH) release assay

To further evaluate cell death, the cell medium was collected and used to measure LDH release with a LDH assay kit (Beyotime Biotechnology, C0016, Shanghai, China). Briefly, the cell medium of primary cortical neurons was collected and centrifuged to remove cell debris after OGD/R and different treatments. The supernatant was mixed with LDH Assay Buffer and Substrate Mix. Subsequently, the absorbance was detected at 490 nm using a microplate reader (BioTek, America). LDH release rate (%) of each group was calculated to be 100% for the non-treatment group.

### Apoptosis assay

Apoptosis was measured using an Annexin V-FITC Apoptosis Detection Kit (Beyotime Biotechnology, C1062L, Shanghai, China) by flow cytometry according to the instructions. HT-22 cells were trypsinized, collected and washed with 1 × PBS after the OGD/R and treatments. 5 × 10^4^ cells of each group were extracted and stained with Annexin V-FITC and PI at room temperature for 20 min in the dark. Apoptosis cell was tested using flow cytometry (Luminex, easyCyte 4th Generation SL System) and analyzed with FlowJo software 10.8.1. Cells that are considered viable are FITC Annexin V and PI negative. In contrast, cells in early apoptosis are FITC Annexin V positive and PI negative, and cells in late apoptosis or already dead are both FITC Annexin V and PI positive. In the present study, the apoptotic cell rate is the total of FITC Annexin V positive and PI negative or both FITC Annexin V and PI positive cells.

### Tunel staining

Cell apoptosis of primary cortical neurons was determined using a commercially available kit (Beyotime Biotechnology, C1089, Shanghai, China) according to the manufacturer’s instructions. Briefly, the primary cortical neurons cultured for 14 days underwent OGD/R and different treatments. All coverslips were fixed with 4% PFA for 30 min and stained with mixed TUNEL solution for 60 min at room temperature. A minimum of three coverslips were used for each experimental group. The number of TUNEL^+^ neurons was evaluated using a microscope (Olympus, Japan) and analyzed using Image J software.

### AAV-9 administration in vivo

The AAV9-PHD2-GFP-shRNA (1.66E + 13 v.g/ml) and AAV9-Con-GFP-shRNA (1.49E + 13 v.g/ml) virus were provided by the Genechem (Shanghai, China) as the sequence of Phd2-mus-1070. The in-site injection of AAV9 followed the previously reported method [24]. Briefly, the C57BL/6JGpt 6-week-old male mice were anesthetized with pentobarbital sodium and fixed in a stereotaxic apparatus (RWD Life Science, 512,600). The virus (1 μl) was injected into the cortex (AP + 0.02 mm; L −3.2 mm; V −1.5 mm) and corpus striatum (AP + 0.5 mm; L −2.0 mm; V −3.0 mm) with a 5-μl syringe (Hamilton) and a 34-gauge needle at 100 nl/min using an injection pump (Micro 4, WPI, Sarasota, Fl, USA). After each injection, the needle was left in place for an additional 10 min and then slowly withdrawn. The virus was allowed to express and knock down the target protein for 4 weeks.

### Prediction of targets of FG4592 and its structural docking

The targets of FG4592 were predicted by the SEA search server [25] [https://sea.bkslab.org/] and Swiss Target Prediction [26] [http://www.swisstargetprediction.ch/]. Moreover, the 2-oxoglutarate-dependent dioxygenases with definite co-crystal structures were also used to dock with FG4592. FG4592 was docked into the co-crystal structures of the PHD2 (Protein Data Bank, PDB, entry 2G19), OGFOD1 (PDB entry 4NHX), FTO (PDB entry 4IE6), JMJD3 (PDB entry 2XUE), UTX/KDM6A (PDB entry 3AVR), MAPK p38ɑ (PDB entry 6Y4T), UTY (PDB entry 3ZLI), ABH1 (PDB entry 6I94), ASPH (PDB entry 6Q9F), JMJD2D (PDB entry 3DXU), JMJD2E (PDB entry 2W2I), KIAA1718 (PDB entry 3KVA), PHF8 (PDB entry 3K3O), PHYHD1 (PDB entry 3OBZ), BBOX (PDB entry 4BGK) using Ledock software [27].

### Analysis of single-cell database online

The online database was used to evaluate the distribution of OGFOD1 in different cell types of the mouse brain (Single cell port: Single Cell Comparison: Cortex data. Data provided by Joshua Levin et al. Systematic comparative analysis of single-cell RNA-sequencing methods. https://singlecell.broadinstitute.org/single_cell/study/SCP425/single-cell-comparison-cortex-data?genes=Ogfod1&cluster=TSNE%20Cortex1%2010X&spatialGroups=--&annotation=CellType--group--study&subsample=all&tab=distribution&distributionPlot=violin&distributionPoints=all#study-visualize).

### Measurement of IC50 in vitro

The half maximal inhibitory concentration (IC50) of FG4592 on KDM6A/UTX or MAPK p38α was measured by the ChemPartner company. The IC50 of FG4592 on JMJD3 was measured by Huawei Pharmaceutical company. Briefly, FG4592 was transferred to the assay plate with serial dilution. FG4592 with different concentrations was added to the substrate solution in a 384-well plate. After incubating and adding the stop solution, the endpoint of the plates of KDM6A/UTX or MAPK p38α was read with EnSpire with Alpha mode or Caliper. The IC50 of FG4592 on JMJD3 was measured by Huawei Pharmaceutical Com. Ltd. All of the enzymatic reactions were conducted in the mixture containing assay buffer, histone H3 peptide substrate, demethylase enzyme, and FG4592 with different concentrations. After enzymatic reactions and adding anti-Mouse Acceptor beads and primary antibody, the samples were measured in an AlphaScreen microplate reader (EnSpire Alpha 2390 Multilabel Reader, PerkinElmer). Enzyme activity assays were performed in duplicates at each concentration, and the A-screen intensity data were analyzed and compared. Brief methods and reports can be provided if needed.

### The hydroxylation of RPS23 with liquid chromatography-mass spectrometry

The HT-22 cells were dealt with vehicle (1 ‰ DMSO in DEME) or 100 μM FG4592 for 24 h. FG4592 was dissolved in DMSO first and diluted in DMEM (the final concentration of DMSO is 1 ‰). The cells were collected by centrifugation and lysed with IP Lysis Buffer (Thermo Scientific^™^ 87,787). The supernatant was incubated with 8 μl anti-RPS23 (Sigma-Aldrich, HPA003215) and mixed overnight with gentle rotation at 4 °C. Then 40 μl Protein A/G PLUS-Agarose (Santa, sc-2003) was added for 4 h at 4 °C. The immunoprecipitates were washed with lysis buffer 3 times and collected. For liquid chromatography-mass spectrometry, all precipitant was tested by the WUHAN Genecreate Biological Engineering Co., LTD. Briefly, the precipitant was degraded by the Trypsin. The mixture of peptides was collected and tested by Q-Exactive HF (Thermo Fisher Scientific, San Jose, CA).

### Immunoblot analysis and immunoprecipitation

According to the previous study [28], the cortical tissues located 1 mm around the infarct border were collected and homogenized in radioimmunoprecipitation (RIPA) lysis buffer (Beyotime, P0013B) containing the phosphatase inhibitor Cocktail I (MCE, Cat. No. HY-K0021) and protease inhibitor cocktail (MCE, Cat. No. HY-K0010). A 40 μg aliquot of protein from each sample was separated using 8%-12% SDS–polyacrylamide gels and then transferred onto PVDF membranes (Millipore, IPFL00010). Membranes were blocked for 1 h at room temperature with 5% non-fat milk in Tris-buffered saline (TBS) before overnight incubation at 4 ℃ with antibodies. The following primary antibodies were used: HIF-1ɑ (1:1,000; abcam, ab179483), HIF-2ɑ (1:1,000; abcam, ab109616), PHD1 (1:1,000; abcam; ab108980), PHD2 (1:3,000; Proteintech; 66589-1-Ig), PHD3 (1:1,000; abcam; ab184714), OGFOD1 (1:1,000; Sigma-Aldrich, HPA003215), RPS23 (1:1,000; Sigma-Aldrich, WH0006228M2), sATF6 (1:1,000; Proteintech, 24169-1-AP), sXBP1 (1:1,000; Proteintech, 24868–1-AP), p-eif2α (1:1,000; Cell Signaling Technology, 3398S), eif-2α (1:1,000; Cell Signaling Technology, 5324S), LC3B (1:1,000; Sigma-Aldrich, L7543), P62/SQSTM1 (1:1,000; Proteintech, 18420-1-AP), Beclin-1 (1:1,000; Proteintech; 11306-1-AP), BCL2 (1:1,000; Proteintech, 12789-1-AP), BAX (1:1,000; Proteintech, 50599-2-LG), cleaved-caspase-3 and caspase-3 (1:3,000; Proteintech, 66470-2-lg), and β-actin (1:2,000; Proteintech, CL594-66009), calreticulin (1:200; proteintech, 27298-1-AP), MAP2 (1:200; abcam, ab11267), GFAP (1:200; proteintech, 60190-1-Ig), Neu (1:200; abcam, ab177487). Secondary antibodies conjugated with HRP against either rabbit or mouse IgG (1:3,000; Proteintech, PR30012 and PR30011) were applied. Immunoreactive bands were visualized and densitometrically analyzed using the Tanon scanner (Tanon, 5200) and Image J software.

HT-22 cells were suspended with the Thermo Scientific™ Pierce™ IP Lysis Buffer (Thermo Scientific^™^ 87787) for immunoprecipitation (IP) after OGD/R. Part of the supernatant (200 μg) was transferred to a new tube as input. For OGFOD1 IP, the supernatant was incubated with 8 μl anti-OGFOD1 (Sigma-Aldrich, HPA003215) and mixed overnight with gentle rotation at 4 °C. Then 40 μl Protein A/G PLUS-Agarose (Santa, sc-2003) was added at 4 °C for 4 h. For RPS23 IP, 60 μl supernatant of HT-22 lysis was incubated with 8 μl anti-RPS23 (Sigma-Aldrich, WH0006228M2) overnight at 4 °C. Then 40 μl of Protein A/G beads were added at 4 °C for 4 h. Subsequently, the immunoprecipitates were washed with lysis buffer 3 times. The immunoprecipitates were then eluted by boiling for 5 min in SDS-PAGE Sample Loading Buffer, 5X (Beyotime, P0015L) and subjected to western blot analysis.

### RNA extraction and real-time PCR

According to the protocol, total RNA from cortical tissue or cells was extracted using the TRIzol (TaKaRa, 9109) method. Briefly, the tissue and cells were homogenized with TRIzol and then mixed with chloroform, and the supernate was extracted gently and mixed with isopropanol. After centrifugation, the sediment was washed with 75% ethanol twice and redissolved by DEPC H_2_O. DNA synthesis was carried out by reverse transcription with equal amounts of RNA (20 ng/μl) using the 4 * gDNA wiper Mix (Vazyme, R233-01) and 5 * qRT SuperMix II (Vazyme, R233-01). qRT-PCR was performed in a 7300 Real-Time PCR System (Applied Biosystems), using 400 ng of cDNA in 2 μl, 0.4 μl of each primer at the concentration, 10 μl of SYBR Green PCR Master Mix (Vazyme, High ROX Premixed, Q141-02) and 7.2 μl DEPC H_2_O. The following programme conditions were applied for qRT-PCR running: 95 ℃ for 5 min followed by 45 cycles at 95 ℃ for 10 s and 60 ℃ for 30 s; melting programme, one cycle at 95 ℃ for 15 s, 60 ℃ for 60 s and 95 ℃ for 15 s. The relative expression of each gene was quantified by the log 2 (−∆∆Ct) method using the gene β-actin as an endogenous control. The primers used in this study are described briefly below: EPO, Forward: ACTCTCCTTGCTACTGATTCCT, Reverse: ATCGTGACATTTT- CTGCCTCC. GLUT1, Forward: CAGTTCGGCTATAACACTGGTG, Reverse: GCCCCCGACA- GAGAAGATG. VEGF, Forward: GCACATAGAGAGAATGAGCTTCC, Reverse: CTCCGCTC- TGAACAAGGCT. PHD2, Forward: GCCGCAGCTCCTTCTACTG, Reverse: TTCATGCACG- GCACGATGTA. OGFOD1: Forward: CGTTCAGCCATGAAGCTATTGC, Reverse: GTTTGG- GATCACACAGTGAAGG, β-actin, Forward: GGCTGTATTCCCCTCCATCG, Reverse: CCAGTTGGTAACAATGCCATGT.

### Immunofluorescence staining

Brains were harvested following cardiac perfusion with ice-cold PBS and 4% PFA and dehydrated using 30% sucrose. All brains were cut into 30 µm thick coronal sections with frozen sections, and cells grown on cover glass were fixed with 4% PFA. All staining samples were permeated with 0.25% Triton X-100 (T109027, Aladdin) at room temperature. After washing the permeabilized cells three times with PBS, the sample was blocked with 0.25% Triton X-100 containing 10% goat serum (ZLI-9056, ZSGB-BIO). After that, the samples were incubated with primary antibodies overnight at 4 °C. Subsequently, the samples were washed three times and incubated with IgG (H + L) Alexa Fluor 488 goat anti-mouse (Invitrogen, A11001) or IgG (H + L) Alexa Fluor 594 goat anti-rabbit (Invitrogen, A11012) secondary antibody for 1 h at room temperature. Finally, the slices and cells were incubated with DAPI (Southern Biotech, USA, CAT No. 0100-20). Images were obtained (594/618 nm for the rabbit Dylight-594-conjugated antibody, 488/519 nm for the mouse Alexa Fluor 488-conjugated antibody) using fluorescence microscopy (A1 HD23, Nikon, Tokyo, Japan). A minimum of three coverslips were used for each experimental group. The area of ER of neurons was analyzed using Image J software according to a method in the previous study [29].

### Statistical analysis

All data from individual experiments were shown as mean ± standard error of the mean (SEM). For the in vitro studies, all experiments were repeated at least three independent times to confirm the results. The results of the in vivo studies were from at least 6 independent mice. For behavioral experiments, n values represented the number of tested mice and were decided considering the minimal use of animals. The sample size of each experimental group/condition is shown in the figure legend. All statistical analyses were performed with GraphPad Prism version 8.0.2 (GraphPad Software, Inc., San Diego, CA). Statistical comparisons were assessed by unpaired Student’s *t*-test or one-way analysis of variance (ANOVA) with Dunnett's *post*-*hoc* test or two-way ANOVA with the Holm-Sidak test for multiple comparisons. Non-parametric data (mNSS) were analyzed with the Mann–Whitney-U test (for two groups) or Kruskal–Wallis test (for multiple groups) followed by Dunn’s *post-hoc*. A *p* < 0.05 was considered statistically significant. Graphs were generated using GraphPad Prism software, too.

## Results

### FG4592 decreases infarct volume and short- or long-term neurologic deficits after transient or permanent brain ischemia

**Fig. 1:**
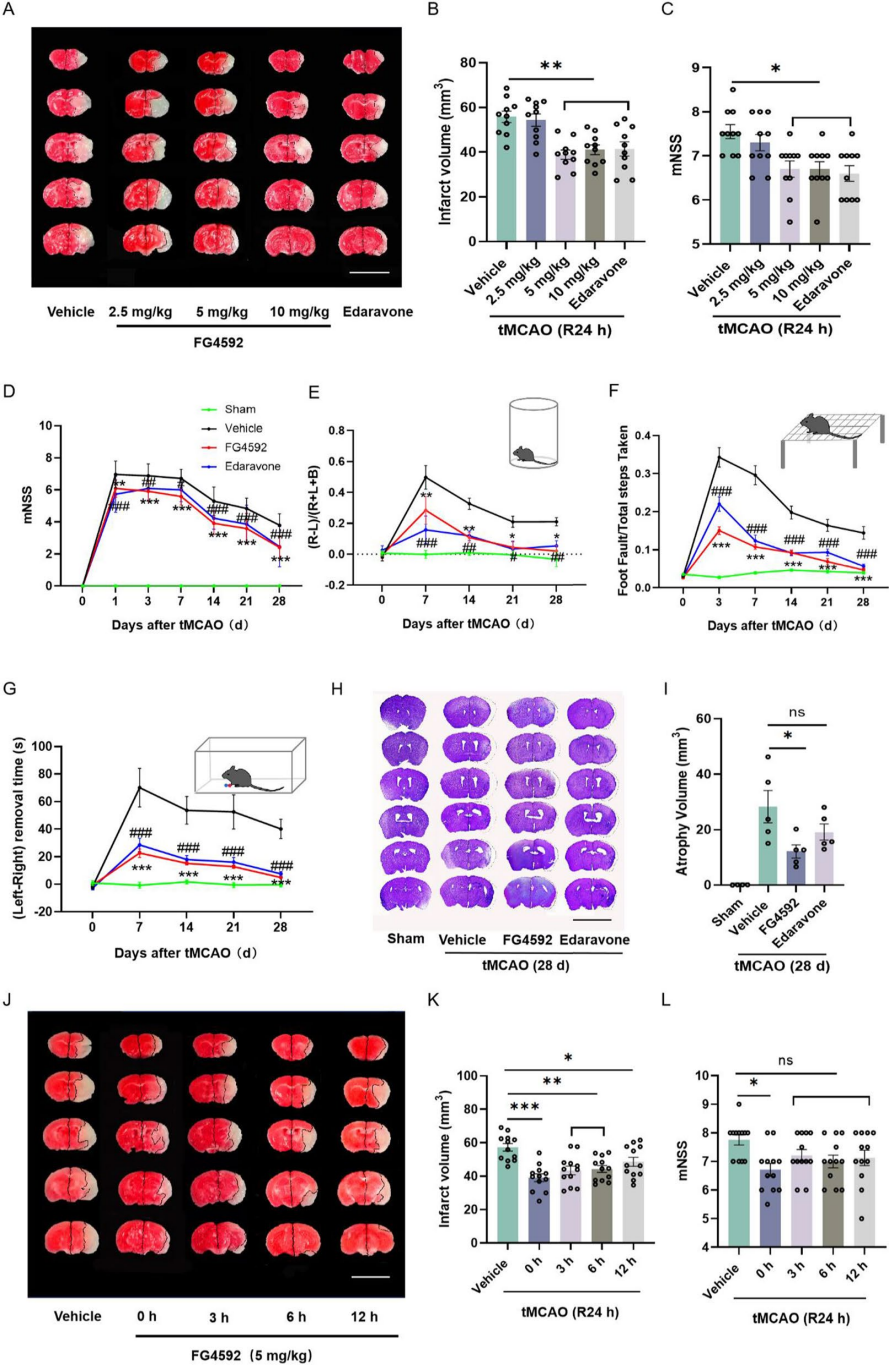
FG4592 alleviates injury and improves the recovery of sensorimotor defects after transient ischemia.** A **Representative TTC staining image of brain sections from mice after 24 h of tMCAO. Layer thickness = 1 mm, scar bar = 1 cm. **B **Infarct volume 24 h after tMCAO in the vehicle (n = 10), FG4592 (2.5 mg/kg, n = 10), FG4592 (5 mg/kg, n = 10), FG4592 (10 mg/kg, n = 10) and Edaravone (3 mg/kg, twice after reperfusion, n = 10) groups. Data are presented as mean ± SEM. Vehicle vs other groups: **p < 0.01 (one-way ANOVA followed by Dunnett’s *post*-*hoc *test). **C **mNSS score of mice after 24h of tMCAO. Data are presented as mean ± SEM. Vehicle vs other groups: *p < 0.05 (non-parametric Kruskal-Wallis test followed by Dunn’s *post-hoc*). **D-G **The sensorimotor functions of mice were analyzed by mNSS score, cylinder test, foot fault task and adhesive removal test up to 28 days after tMCAO. Data are presented as mean ± SEM. FG4592 (n = 11) vs vehicle (n = 12): ***p < 0.001, **p < 0.01, *p < 0.05. Edaravone (n = 11) vs vehicle: ###*p* < 0.001, ##*p* < 0.01, #*p* < 0.05 (two-way repeated-measures ANOVA followed by Holm-Sidak *post-hoc* multiple-comparison tests).** H **The representative crystal violet staining images correspond to coronal brain sections from mice after 28 days of tMCAO. Five mice were randomly selected from each group after the behaviour tests. Scar bar = 1cm.** I **Measurement of the atrophy volume of the infarct hemisphere. Vehicle vs FG4592: *p < 0.05, ns: no significance (one-way ANOVA followed by Dunnett’s *post*-*hoc *test, n = 5).** J **The representative TTC staining images of mice administrated with FG4592 at different time points after tMCAO. Layer thickness = 1 mm, scar bar = 1 cm. **K **and **L** The infarct volumes (one-way ANOVA followed by Dunnett’s *post*-*hoc *test) and mNSS scores (non-parametric Kruskal-Wallis test followed by Dunn’s *post-hoc*) of the vehicle (n = 12), 0-hour (n = 12), 3 hours (n = 12), 6 hours (n = 12) and 12 hours (n = 12) groups. Data are presented as mean ± SEM. vehicle vs other groups: ***p < 0.001, **p < 0.01, *p < 0.05, ns: no significance

To determine whether FG4592 can alleviate brain injury in mice subjected to tMCAO or dMCAO. The infarct volumes and behavioral deficits of mice were evaluated 1 day after the stroke (Additional file 1: Fig. S1A). Compared with the CBF of baseline, mice with a CBF reduction of at least 75% were used for the subsequent experiments (Additional file 1: Fig. S1B, C). As shown in Fig. 1A–C, FG4592 (5 or 10 mg/kg) significantly decreased the infarct volumes and mNSS score after 24 h of reperfusion compared with the vehicle group, which was not inferior to the edaravone. Moreover, FG4592 treatment resulted in a remarkable recovery in somatosensory and locomotor functions (Fig. 1D–G) after tMCAO, which showed a neuroprotective effect similar to edaravone. In addition, FG4592 prevented brain atrophy in mice after 28 days of stroke (Fig. 1H–I). There was not any difference in the survival rate of mice among all groups after tMCAO (Additional file 1: Fig. S2A). As recommended by STAIR guidelines, drugs with wider therapeutic window would have more translational value [12, 13]. Herein, FG4592 was administrated 3, 6 and 12 h after tMCAO to verify the therapeutic window of stroke. Delayed administration of FG4592 also significantly reduced the infarct volumes at all time points (Fig. 1J, K). However, a significant mNSS decline was only observed in the 0-h group after reperfusion, while a slightly reduced trend of mNSS was observed for the other time points (Fig. 1L). Even in the dMCAO mice models, FG4592 decreased infarct volumes (Additional file 1: Fig. S2B and C) and short-term or long-term neurological deficits (Additional file 1: Fig. S2D–H). According to STAIR recommendations, both female, old mice (at least 10 months) and mice with comorbidity were included in the present study [12]. The equal protective effect of FG4592 was observed similarly in aged, female, or DIO mice assessed by infarct volumes and mNSS (Additional file 1: Fig. S3A–K). These findings suggested that FG4592 can alleviate the ischemic injury and promote the recovery of neurological deficits in male, female, and aged or DIO mice after transient or permanent ischemic.

**Fig. 2:**
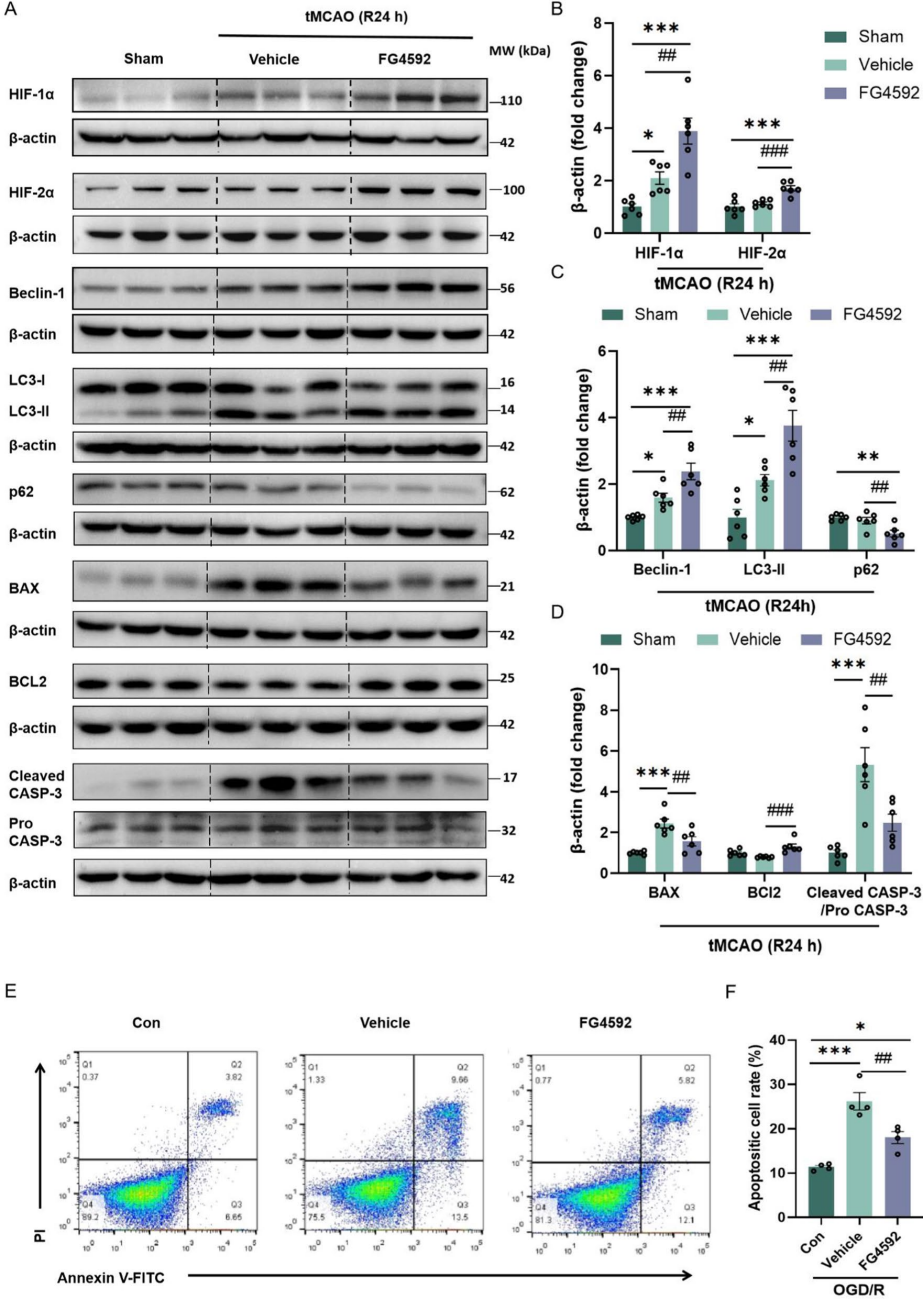
FG4592 activates autophagy and inhibits the apoptotic pathway after I/R injury. **A** Western blot images of HIF-1ɑ, HIF-2ɑ, Beclin-1, LC3-II, p62/SQSTM1, BAX, BCl2, cleaved-caspase-3 and caspase-3 in the peri-infarct brain tissues of Sham, vehicle and FG4592 groups after 24 hours of tMCAO. **B-D** Immunoblot analyses of proteins are shown in panel A. Data are presented as mean ± SEM. Sham vs vehicle or FG4592: ***p < 0.001, **p < 0.01, *p < 0.05. vehicle vs FG4592: ###*p *< 0.001, ##*p* < 0.01 (one-way ANOVA followed by Dunnett’s *post*-*hoc *test, n = 6 in each group). **E **and **F** Apoptotic cell analyses of HT-22 cells were detected using an Annexin V-FITC apoptosis flow cytometry. Cells underwent OGD for 3 hours and were treated with FG4592 or vehicle for 6 hours after reperfusion. The fractions of apoptotic cells were semi-quantified by Flowjo software. Data are presented as the mean ± SEM and from four independent experiments. Sham vs vehicle or FG4592: ***p < 0.001, *p < 0.05. vehicle vs FG4592: ##*p* < 0.01 (one-way ANOVA followed by Dunnett’s *post*-*hoc *test)

As its classical pharmacological effects, FG4592 also upregulated the HIF signal pathway in the peri-infarct area of mice after ischemia. Compared with the sham and vehicle groups, FG4592 increased the protein levels of HIF-1/2ɑ (Fig. 2A, B). Autophagy and apoptosis are crucial pathophysiological processes in ischemic stroke. Previous studies have indicated that the appropriate activation of autophagy and inhibition of apoptosis benefit brain recovery after ischemia–reperfusion (I/R) injury [30, 31]. Compared with the vehicle group, FG4592 promoted the expression of LC3-II and Beclin-1 but down-regulated the expression of p62 in peri-infarct tissue (Fig. 2A, C). Moreover, mice treated with FG4592 showed lower BAX and cleaved-caspase-3 and higher levels of BCL2 after tMCAO (Fig. 2A, D). Consistent with the in vivo results, FG4592 reduced the apoptotic cell rate of HT-22 cells after OGD/R (Fig. 2E, F). All data indicate that FG4592 activates the autophagy flux pathway and inhibits cellular apoptosis after I/R injury.

### FG4592 reduces ischemic-reperfusion injury in a HIF-independent manner in vitro and in vivo

**Fig. 3:**
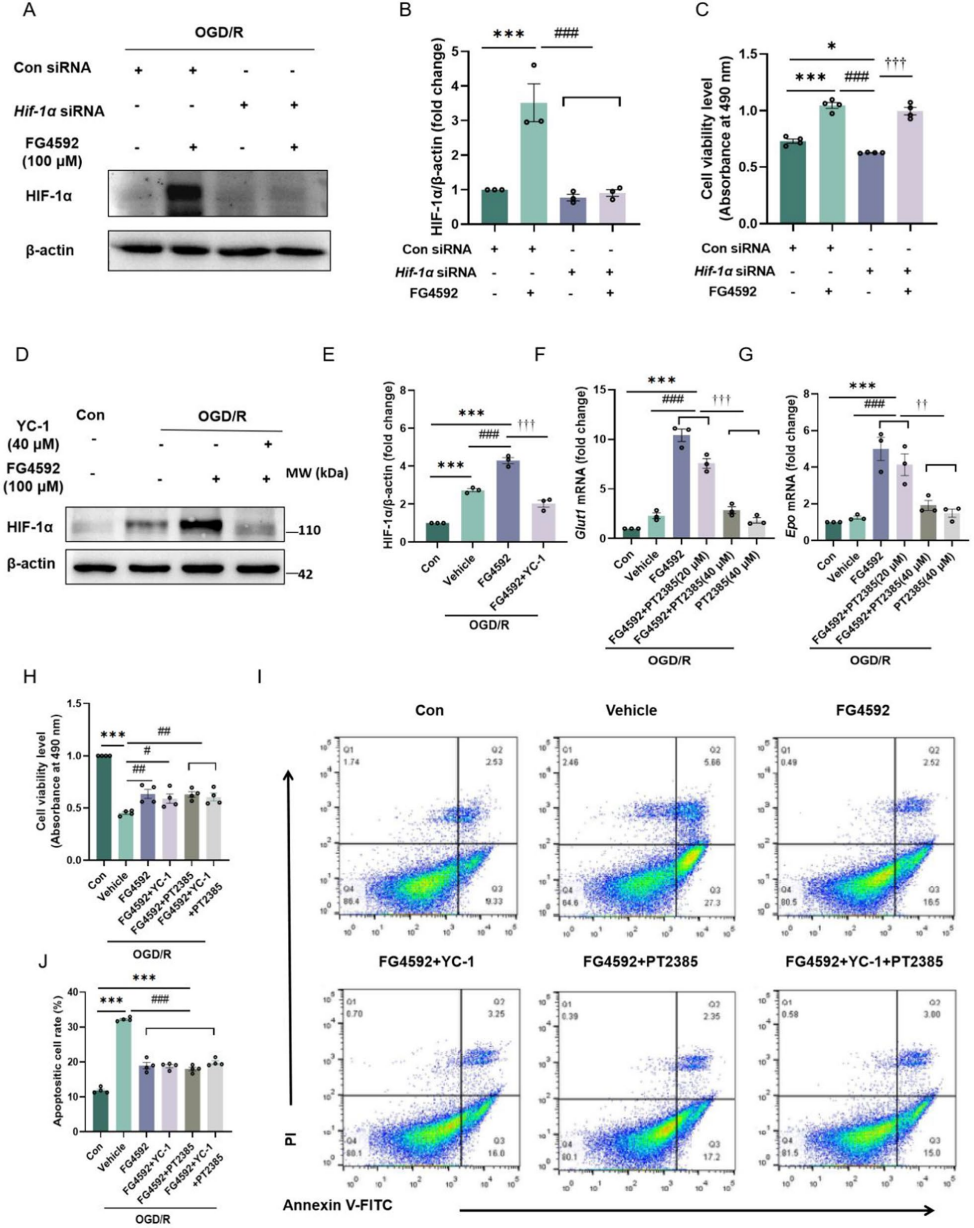
FG4592 exerts a neuroprotective effect in a HIF-α independent manner in vitro after I/R injury. **A **and **B** Representative immunoblot and analysis of HIF-1ɑ in HT-22 cells transfected with con siRNA or *Hif-1α* siRNA for 48 hours and further treated with FG4592 after OGD. Data are presented as the mean ± SEM, Con siRNA vs Con siRNA+FG4592: ***p < 0.001, Con siRNA+FG4592 vs *Hif-1α *siRNA or *Hif-1α *siRNA+FG4592: ###*p* < 0.001 (one-way ANOVA followed by Dunnett’s *post*-*hoc *test, n = 3). **C** The cell viability of HT-22 cells was measured by the absorbance of formazan products following OGD/R. Results are presented as mean ± SEM from four independent experiments, Con siRNA vs Con siRNA+FG4592 or *Hif-1α *siRNA: ***p < 0.001, *p < 0.05. Con siRNA+FG4592 vs *Hif-1α *siRNA: ###*p* < 0.001. *Hif-1α *siRNA vs *Hif-1α *siRNA+FG4592: †††*p* < 0.001 (one-way ANOVA followed by Dunnett’s *post*-*hoc *test). **D **and **E** Immunoblot and analysis of HIF-1ɑ in HT-22 cells. The cells of YC-1 groups were pretreated with YC-1 before 1 hour of OGD/R. All experimental groups underwent OGD for 3 hours and were treated with vehicle or FG4592 for 6 hours after reperfusion. **F **and **G** The relative expressions of *Glut1* and *Epo *mRNA in HT-22 cells after OGD/R. The cells of PT-2385 pretreated groups were first dealt with PT-2385 for 1 hour before OGD/R. All experimental groups underwent OGD/R for 3 hours and were treated with vehicle or FG4592 for 6 hours after OGD/R. Data are presented as the mean ± SEM and from three independent experiments. Con vs other groups: ***p < 0.001, **p < 0.01, vehicle vs other groups: ###*p* < 0.001, FG4592 or FG4592+PT2385 (20 μM) vs FG4592+PT2385 (40 μM) or PT2385 (40 μM): †††*P *< 0.001 (one-way ANOVA followed by Dunnett’s *post*-*hoc *test, n = 3). **H** The cell viability of HT-22 cells was measured by the absorbance of formazan products following OGD/R. The cells of YC-1 and/or PT-2385 pretreated groups were first dealt with YC-1 and/or PT-2385 for 1 hour before OGD/R. All experimental groups underwent OGD/R for 3 hours and were treated with vehicle or FG4592 for 6 hours after OGD/R. Results are presented as mean ± SEM from four independent experiments, Con vs vehicle: ***p < 0.001. vehicle vs other groups: ##*p* < 0.01, #*p* < 0.05. **I **and **J **Apoptotic cell rates of HT-22 cells after OGD/R were examined by Annexin V-FITC/PI-labeled flow cytometry. Data are expressed as mean ±SEM of four independent experiments. Con vs other groups: ***p < 0.001. vehicle vs other groups: ###*p* < 0.001 (one-way ANOVA followed by Dunnett’s *post*-*hoc *test)

Previous studies have shown that other PHDs inhibitors play neuroprotective functions in rodents after ischemic stroke, whether administered pre-stroke or post-stroke [8]. It is interesting to note that the neuroprotective effects of some PHDs inhibitors are not HIF-1α dependent [32–34]. However, the specific mechanisms of this protection are still unknown. Therefore, blocking the expression or function of HIF-1ɑ or HIF-2ɑ in FG4592-treated cells or mice could provide further evidence to settle this controversy. In the present study, *Hif-1α* siRNA inhibited expression of Hif-1α. As shown in Fig. 3A, B, *Hif-1α* siRNA could inhibit the expression of HIF-1ɑ induced by FG4592 after I/R injury. Meanwhile, the knockdown of HIF-1ɑ did not attenuate the ischemia protection of FG4592 (Fig. 3C).

YC-1 can inhibit the expression of HIF-1ɑ protein induced by hypoxia and has been used to be a potential antitumor candidate [35, 36]. PT2385 can specifically block the dimerization of HIF-2α and HIF-1β, which inhibits the expression of HIF-2α-dependent genes like *Epo* and *Glut1* [37]. In the present study, administration of YC-1 (40 μM) before OGD/R inhibited the expression of HIF-1ɑ in HT-22 cells treated with FG4592 after I/R (Additional file 1: Fig. S4A and B, Fig. 3D, E). Moreover, pretreatment with PT2385 (40 μM) down-regulated the expression of *Epo* and *Glut1* mRNA induced by FG4592 (Fig. 3F, G). As shown by the cell viability analysis after OGD/R, the protective function of FG4592 was not abolished by single or combined pretreatment of YC-1 and PT2385 (Fig. 3H). Moreover, compared with the FG4592 group, the apoptotic cell rates did not increase in the group subjected to single or combined pretreatment with YC-1 and PT2385 (Fig. 1I, J).

**Fig. 4:**
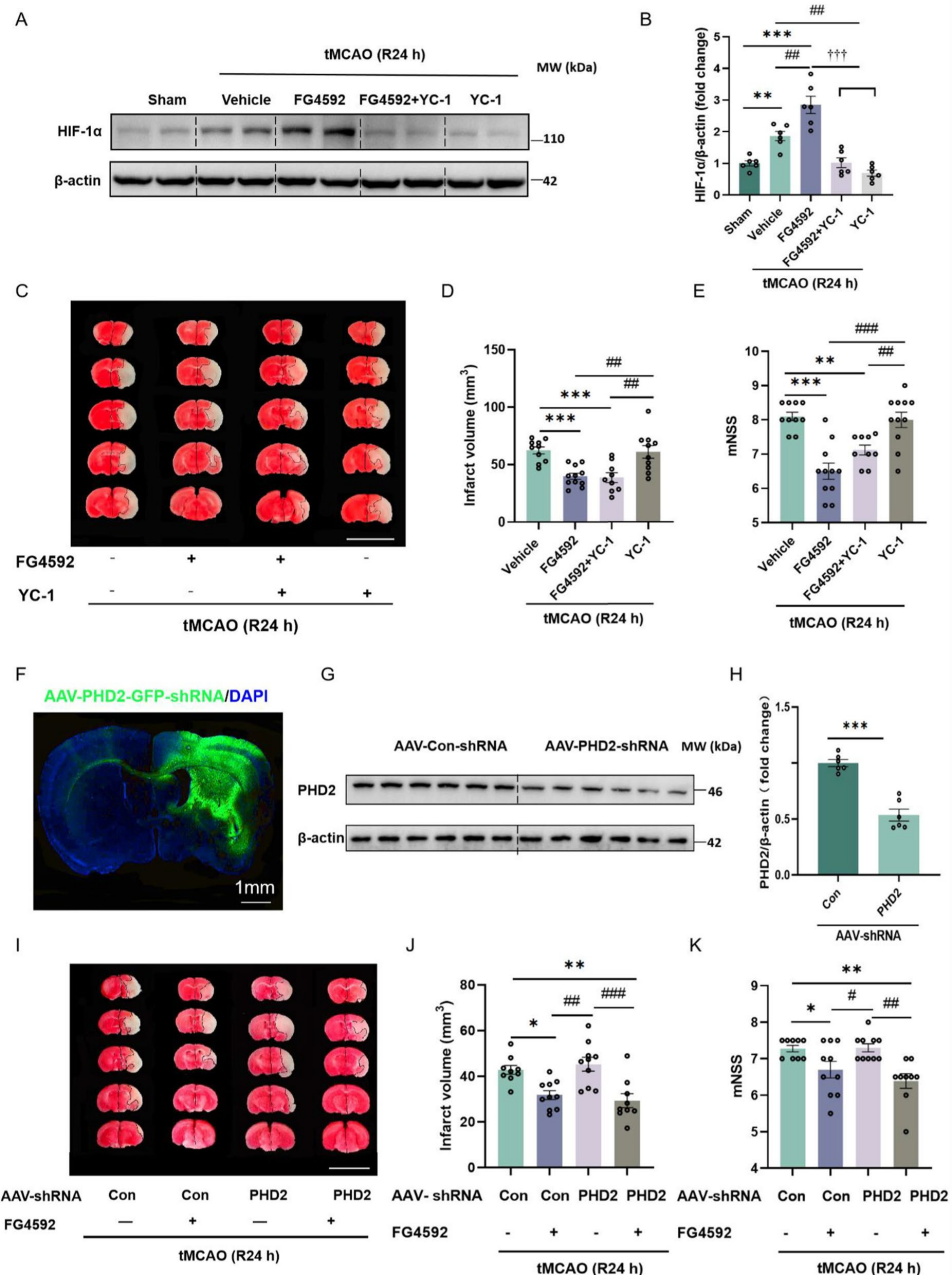
FG4592 maintains a neuroprotective effect in a HIF-α independent manner in vivo after I/R injury. **A **and **B **Representative western blots and statistical analyses of band intensity of HIF-1ɑ protein in peri-infarct brain tissue of mice after 24 h of tMCAO. The mice of YC-1 pretreated groups were injected with YC-1 (10 mg/kg,* ip*) for 1 hour before tMCAO. All experimental groups were subjected to tMCAO and treated with FG4592 or vehicle after 5 min of reperfusion. Data are presented as mean ± SEM. Sham vs vehicle or FG4592: ***p < 0.001, **p < 0.01. vehicle vs FG4592 or FG4592+YC-1 or YC-1: ##*p* < 0.01. FG4592 vs FG4592+YC-1 or YC-1: †††*p* < 0.001 (one-way ANOVA followed by Dunnett’s *post*-*hoc *test, n=6). **C** Representative images of brain TTC staining from mice in each group. Layer thickness = 1 mm, scar bar = 1 cm. **D **and **E **The infarct volumes (one-way ANOVA followed by Dunnett’s *post*-*hoc *test) and mNSS scores (non-parametric Kruskal-Wallis test followed by Dunn’s *post-hoc*) of the vehicle (n = 10), FG4592 (n = 11), FG4592+YC-1 (n = 9) and YC-1 groups (n = 10) after 24 h of tMCAO. Data are presented as mean ± SEM. vehicle vs FG4592 or FG4592+YC-1: ***p < 0.001, **p < 0.01. YC-1 vs FG4592 or FG4592+YC-1: ###*p* < 0.001, ##*p* < 0.01. **F** Fluorescent staining of mouse brains injected with AAV-PHD2-shRNA in situ for 28 d (Left: 4×, Scale bar, 1 mm). **G **and **H** Representative western blot and protein level of PHD2 in mouse brains. All mice were cortical in situ injected with AAV-PHD2-GFP-shRNA or AAV-Con-GFP-shRNA virus for 28 days (n = 6 in each group). Data are presented as mean ± SEM. AAV-Con-shRNA vs AAV-PHD2-shRNA: ***p < 0.001 (student’s *t*-test. n = 6). **I **Representative images of TTC-stained brain slices of mice after 24 hours of tMCAO. All mice were cortical in situ injected with AAV-Con-shRNA or AAV-PHD2-shRNA for 28 days and underwent tMCAO. Layer thickness = 1 mm, scar bar = 1 cm. **J **and **K** The quantitative infarct volume and mNSS of AAV-Con-shRNA (n = 9), AAV-Con-shRNA+FG4592 (n = 10), AAV-PHD2-shRNA (n = 10) and AAV-PHD2-shRNA + FG4592 (n = 9) groups after 24 hours of tMCAO. Data are presented as mean ± SEM, AAV-Con-shRNA vs AAV-Con-shRNA+FG4592 or AAV-PHD2-shRNA+FG4592: **p < 0.01, *p < 0.05. AAV-PHD2-shRNA vs AAV-Con-shRNA + FG4592 or AAV-PHD2-shRNA+ FG4592: *###p *< 0.001, #*#p *< 0.01, *#p *< 0.05 (one-way ANOVA followed by Dunnett’s *post*-*hoc *test for infarct volume, non-parametric Kruskal-Wallis test followed by Dunn’s *post-hoc *for mNSS)

In the peri-infarct tissue of mice after tMCAO, the pretreatment with YC-1 down-regulated the increase of HIF-1ɑ protein induced by FG4592 (Fig. 4A, B). However, compared with the FG4592 group, the pretreatment with YC-1 did not alleviate the neuroprotective function of FG4592, based on infarct volume and mNSS (Fig. 4C–E). Moreover, the increased autophagy and decreased apoptotic level in FG4592-treated mice brains were not attenuated by the pretreatment with YC-1 (Additional file 1: Fig. S5A-G). Altogether, these results indicate that the therapeutic effects of FG4592 may not depend only on HIF-α.

As the main isoform of PHDs regulating HIF-ɑ, PHD2 is the critical target of FG4592 in the central nervous system [38]. Accordingly, we constructed AAV-PHD2-shRNA based on the siRNA sequence verified in vitro (Additional file 1: Fig. S6A-C). The AAV-PHD2-shRNA can infect the cortex and corpus striatum after in situ injection (Fig. 4F). Compared with AAV-Con-shRNA, the PHD2 protein level in the cortex of the AAV-PHD2-shRNA group was notably reduced after 28 days of injection (Fig. 4G–H). One month after injection of the virus, down-regulation of PHD2 alone did not decrease the infarct volumes and mNSS score after 24 h of tMCAO. Furthermore, the neuroprotective effect of FG4592 was not abolished by the decreased expression of PHD2 in mouse brains after ischemic injury (Fig. 4I–K). All these data suggested that the neuroprotective function of FG4592 is not or not only dependent on the PHD2 or HIF.

### FG4592 binds to the novel pharmacological target OGFOD1 and inhibits the prolyl hydroxylation of RPS23 within neurons

**Fig. 5:**
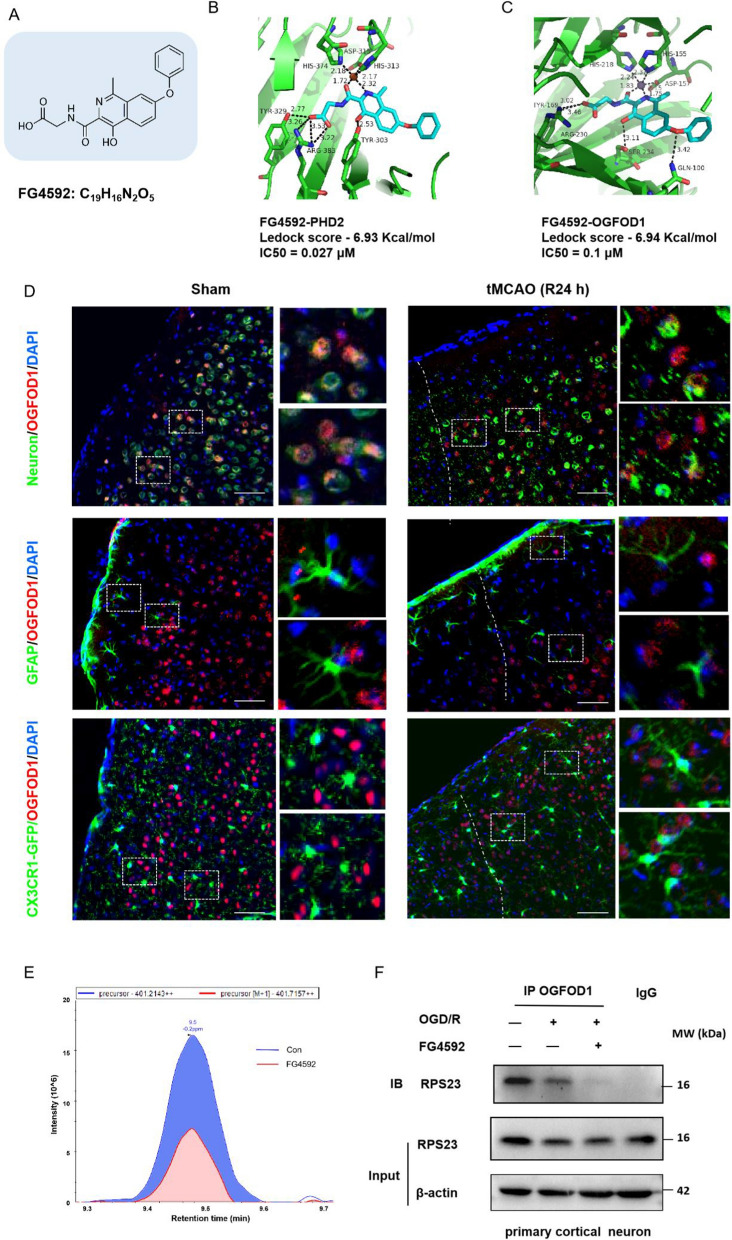
FG4592 inhibits the enzymatic activity of OGFOD1 and blocks its combination and hydroxylation of RPS23. **A** Molecular structure of FG4592. **B **and **C** Docking analyses of FG4592 covalent binding modes to PHD2 and OGFOD1. **D** The fluorescent staining images of OGFOD1 and different neural cell markers in healthy brains of mice or peri-infarct brains of mice after 24 hours of tMCAO. The NeuN and GFAP were markers of neurons and astrocytes, respectively. The brain slices of CX3CR1-GFP mice were used to exhibit the microglia. Scale bar, 100 μm, 40 ×. **E** The hydroxylation area of Pro-62 within RPS23 immunoprecipitated. Cells were dealt with vehicle or 100 μM FG4592 for 24 hours, and its split product was immunoprecipitated by anti-RPS23.** F** Representative western blots of RPS23 in the anti-OGFOD1 immunoprecipitates and input groups. Primary neuron cells underwent OGD for 3 hours and were treated with vehicle or FG4592 for 6 hours after reperfusion. The cell extracts were immunoprecipitated with OGFOD1 antibody or control IgG and immunoblotted for RPS23

Considering more candidate targets of FG4592, online predicting and molecular docking were used to explore the potential targets of FG4592 based on its structure (Fig. 5A. Additional file 2: Tables S1-S3). Based on the measurement of IC50 in vitro research and previous studies, FG4592 can inhibit the activity of OGFOD1 [39], UTX/KDM6A, and FTO [40] besides its classical target PHD2 (Fig. 5B, C. Additional file 1: Fig. S7A–I). According to previous research, OGFOD1 is another type of 2OG-dependent non-heme iron dioxygenases and has high homogeneity in its structure-based sequence and activating site with PHD2 [41]. According to a comprehensive analysis of the Ledock score and IC50 [39], OGFOD1 is a novel potential target of FG4592 (Fig. 5C). OGFOD1 is highly expressed in the brain, based on evidence from online databases and a prior study [42, 43]. According to the online database of the single-cell sequence data, OGFOD1 is mainly found in excitatory and inhibitory neurons (Additional file 1: Fig. S7J). To confirm the distribution of OGFOD1 in mouse brains, we performed immunofluorescence analysis using NeuN, GFAP and CX3CR1-GFP as markers for neurons, astrocytes and microglia, respectively. The results showed that OGFOD1 was located in the neurons of mouse brains with and without ischemic stroke (Fig. 5D). To determine whether FG4592 can alleviate the ischemic injury of primary cortical neurons after OGD/R, the cell viability was determined by CCK-8 and LDH leakage assay. Cell viability was observed to be significantly decreased, and the level of LDH release was remarkably high in the primary cortical neurons of the vehicle group. However, FG4592 significantly increased cell viability and decreased LDH release (Additional file 1: Fig. S8A and B). Moreover, TUNEL staining was used to assess the apoptotic cells in the primary cortical neurons of mice after OGD/R. The co-staining and quantitative results showed that TUNEL-positive neurons were markedly reduced in the FG4592 treated group (Additional file 1: Fig. S8C and D). All in vitro results indicated the protective effect of FG4592 against primary cortical neurons from ischemic injury.

Previous studies have reported that OGFOD1 can combine with small ribosomal protein s23 (RPS23) to hydroxylate the Pro-62 of RPS23, which modulates protein translation and splicing [44, 45]. Representative confocal immunofluorescence images showed that OGFOD1 and RPS23 are co-located in the primary cortical neurons (Additional file 1: Fig. S9A). The LC–MS/MS analysis revealed hydroxylation of Pro-62 within RPS23 in Anti-RPS23 immunoprecipitates of HT-22 cells which were treated with vehicle or FG4592 (100 μM) (Additional file 1: Fig. S9B and C). Post-translational protein modification analysis indicated that hydroxylation of RPS23 decreased in FG4592-treated cells (Fig. 5E, Additional file 2: Table S4). Moreover, compared with the vehicle group, the protein level of RPS23 was reduced in Anti-OGFOD1 immunoprecipitates of primary cortical neurons treated with FG4592 following OGD/R (Fig. 5F). Together, these results demonstrated that FG4592 could inhibit the hydroxylation of Pro-62 within RPS23 by targeting OGFOD1.

### Inhibiting OGFOD1 prevents cell injury and activates the unfolded protein response and autophagy after I/R injury

**Fig. 6:**
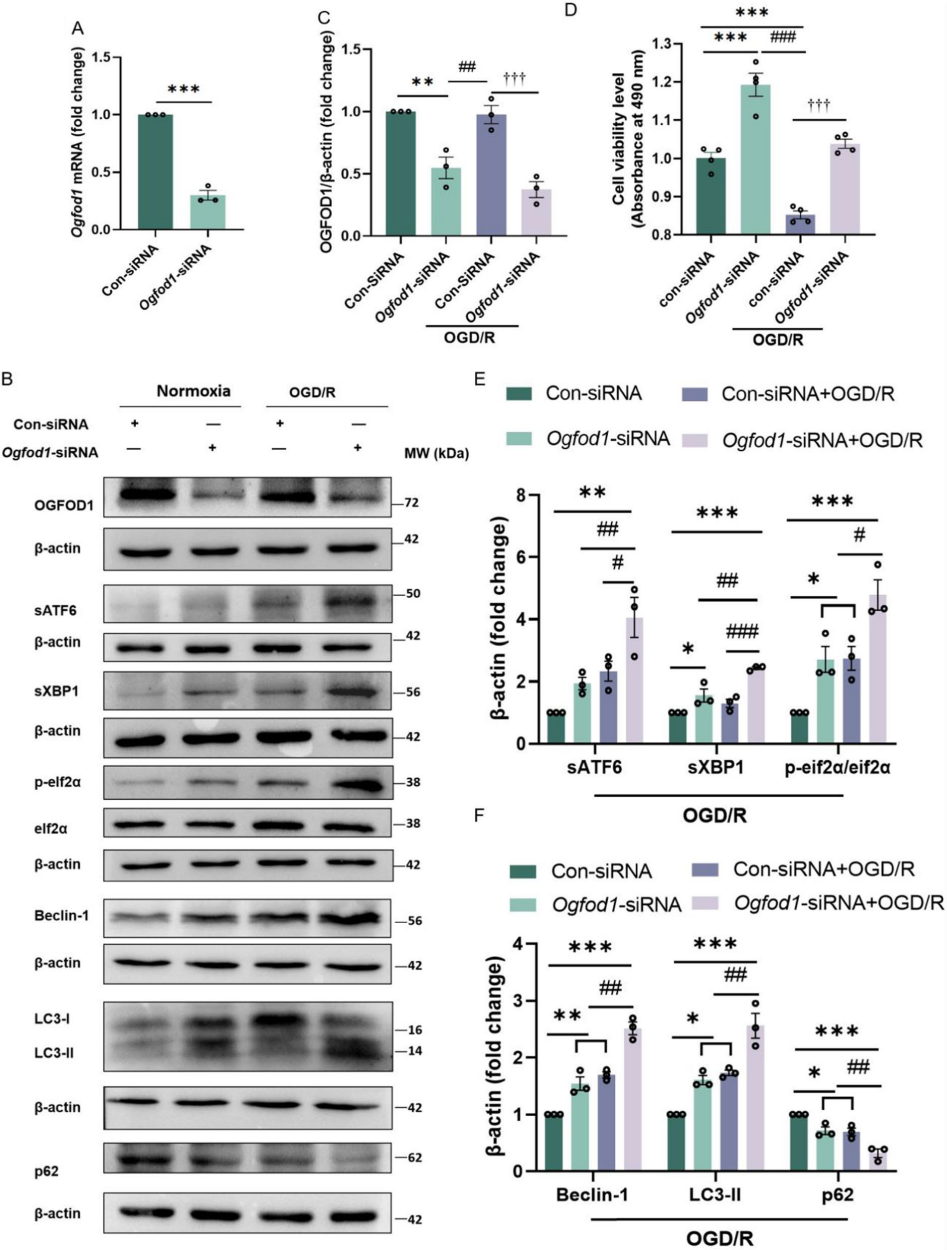
OGFOD1 knockdown activates UPR and protects cells against I/R injury.** A **The mRNA level of *Ogfod1 *in HT-22 cells transfected with Con-siRNA and *Ogfod1-*siRNA for 36 hours previously. Con-siRNA vs *Ogfod1-*siRNA: ***p < 0.001 (student’s *t*-test. n = 3). **B **Representative western blots of OGFOD1, sATF6, sXBP1, p-eIF2ɑ, Beclin-1, LC3-II and p62/SQSTM1 in HT-22 cells. Cells were transfected with Con-siRNA or *Ogfod1-*siRNA for 48 hours and underwent OGD (3 hours)/R (6 hours). **C **The quantitative results of OGFOD1 in HT-22 cells transfected with con-siRNA or *Ogfod1-*siRNA for 48 hours and subjected to OGD/R. Data are presented as the mean ± SEM, Con-siRNA vs *Ogfod1*-siRNA: **p < 0.01, *Ogfod1*-siRNA vs *Ogfod1*-siRNA + OGD/R: ##*p* < 0.01, *Ogfod1*-siRNA+OGD/R vs Con-siRNA+OGD/R: †††*p* < 0.001 (one-way ANOVA followed by Dunnett’s *post*-*hoc *test, n = 3). **D **The cell viability of HT-22 cells was measured by the absorbance of formazan products following OGD/R. Cells were transfected with Con-siRNA or *Ogfod1-*siRNA for 48 hours and underwent OGD (3 hours)/R (6 hours). Results are presented as mean ± SEM from four independent experiments, Con-siRNA vs *Ogfod1*-siRNA or *Ogfod1*-siRNA+OGD/R: ***p < 0.001, *Ogfod1*-siRNA vs *Ogfod1*-siRNA+OGD/R: ###*p* < 0.001, *Ogfod1*-siRNA+OGD/R vs Con-siRNA+OGD/R: †††*p* < 0.001 (one-way ANOVA followed by Dunnett’s *post*-*hoc *test). **E **and **F **The quantitative results of sATF6, sXBP1, p-eIF2ɑ, Beclin-1, LC3-II and p62/SQSTM1 in HT-22 cells transfected with Con-siRNA or *Ogfod1-*siRNA for 48 hours and subjected to OGD/R. Data are presented as the mean ± SEM, Con-siRNA vs other groups: ***p < 0.001, **p < 0.01, *p < 0.05. *Ogfod1*-siRNA+OGD/R vs *Ogfod1*-siRNA or Con-siRNA+OGD/R: ###*p* < 0.001, ##*p* < 0.01, #*p* < 0.05 (one-way ANOVA followed by Dunnett’s *post*-*hoc *test, n = 3)

As a 2-OG-dependent oxygenase, the knockdown of *Ogfod1* promotes the up-regulation of critical proteins associated with the unfolded protein response, such as p-eif2ɑ and sXBP1 [44, 46]. According to a prior study, the UPR pathway was upregulated in the heart tissue of mice with *Ogfod1* knockout after the I/R injury [47]. Moreover, it has been reported that activating UPR with drugs or genetic animal models showed a neuroprotective role in ischemic stroke models [48]. Herein, the *Ogfod1* siRNA significantly inhibited the expression of *Ogfod1* mRNA and its protein level (Fig. 6A–C). To determine the neuroprotection of inhibiting OGFOD1, the cell viability of HT-22 cells transfected with *Ogfod1* siRNA was measured. As shown in Fig. 6D, the knockdown of OGFOD1 decreased cell injury after OGD/R. We further measured the expression of the crucial proteins of the UPR pathway. As shown in Fig. 6B, E, the expression of sATF6, sXBP1 and p-eif2α in cells transfected with *Ogfod1* siRNA were increased than the Con-siRNA group after the OGD/R.

Activation of the UPR triggers autophagy, ER-associated degradation (ERAD) and the shutdown of translation, which decreases the burden of unfolded or overloaded proteins and mitigates cell injury after stress [48]. The modest activation of autophagy can alleviate ischemic cells or brain injury after the ischemic stroke [30]. To determine whether inhibiting OGFOD1 can activate autophagy. The autophagy-related proteins were measured in HT-22 cells transfected *Ogfod1* siRNA. The upregulations of LC3-II, Beclin-1 and downregulation of p62 were induced by the transfection of *Ogfod1*-siRNA after OGD/R (Fig. 6B, F). All these data indicated that selective inhibition of OGFOD1 can increase UPR and alleviate ischemic injury in vitro after OGD/R. Moreover, inhibiting OGFOD1 also promoted cellular autophagic levels after the I/R injury.

### FG4592 promotes UPR and autophagic flux in primary cortical neurons by inhibiting OGFOD1 after I/R injury

**Fig. 7:**
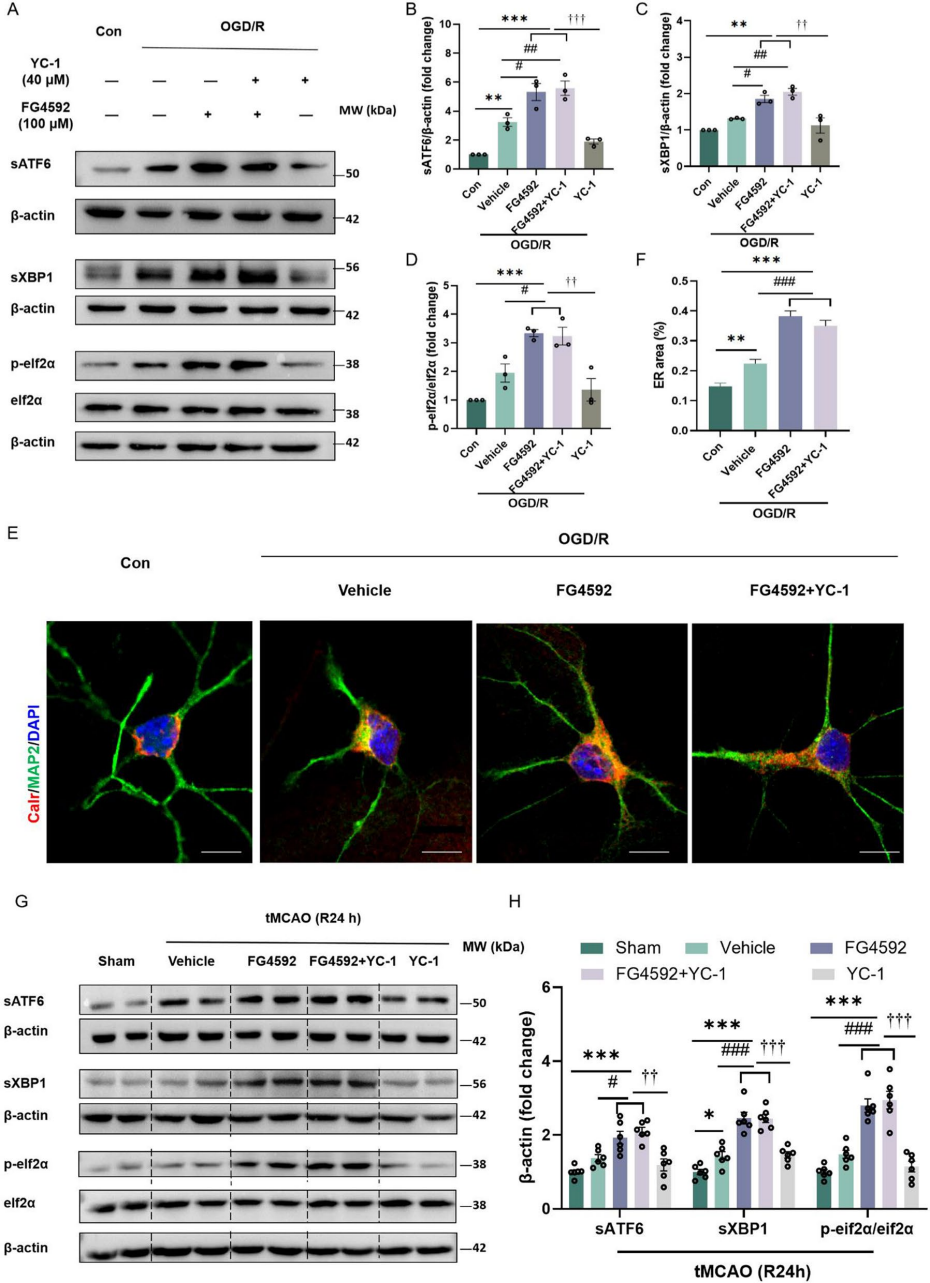
FG4592 also activates the UPR in a HIF-1α independent way in vitro and in vivo. **A-D** Immunoblots and quantitative analyses of sATF6, sXBP1 and p-eIF2ɑ in primary cortical neurons. Cells of YC-1 groups were pretreated with YC-1 for 1 hour previously. All experimental groups were treated with OGD for 3 hours and then treated with vehicle or FG4592 after reperfusion. Data are presented as the mean ± SEM, Con vs other groups: ***p < 0.001, **p < 0.01, vehicle vs FG4592: ##*p* < 0.01, #*p* < 0.05, FG4592 or FG4592+YC-1 vs YC-1: †††*p* < 0.001, ††*p* < 0.001 (one-way ANOVA followed by Dunnett’s *post*-*hoc *test, n = 3). **E** Representative confocal immunofluorescence images labelled with the ER marker calreticulin and the MAP2 in the primary cortical neurons. Scale bar = 10 μm. **F **The quantitative analysis of the ER area of different groups. Data are presented as mean ± SEM and were randomly from three independent coverslips. Con vs other groups: ***p＜0.001, **p＜0.01. Vehicle vs FG4592 or FG4592+YC-1: ###*p* < 0.001 (one-way ANOVA followed by Dunnett’s *post*-*hoc *test, n = 40 neurons in each group). **G** The representative western blots of sATF6, sXBP1 and p-eif2α in peri-infarct tissue of mice after 24 hours of tMCAO. Mice pretreated with YC-1 were injected with YC-1 intraperitoneally 1 hour before tMCAO. The experimental groups were treated with vehicle or FG4592 after tMCAO. **H** The statistical analysis of sATF6, sXBP1 and p-eif2α in the G panel. Data are presented as mean ± SEM. Sham vs other groups: ***p < 0.001, *p < 0.05. vehicle vs FG4592 or FG4592+YC-1 group: ##*#p* < 0.001, *#p* < 0.05. YC-1 vs FG4592 or FG4592+YC-1 group: †††*p* < 0.001, ††*p* < 0.01 (one-way ANOVA followed by Dunnett’s *post*-*hoc *test, n = 6 in each group)

As mentioned above, FG4592 can inhibit PHDs and OGFOD1 simultaneously, and OGFOD1 inhibition could affect the UPR and autophagy and protect cells against ischemic injury. However, whether FG4592 can activate the UPR following an ischemic stroke remains unclear. As shown in Fig. 7A–D, the protein levels of sATF6, sXBP1 and p-eif2ɑ were significantly increased in primary cortical neurons treated with FG4592 after OGD/R. However, a prior study found that HIF-1α activates the UPR pathway in *Phd2*-deficient chondrocytes [49]. To determine whether FG4592 can activate the UPR by inhibiting OGFOD1 but not upregulating HIF-1α after I/R injury. Primary cortical neurons were pretreated with YC-1. Notably, the pretreatment of YC-1 did not impede the expression of sATF6, sXBP1, and p-eif2ɑ induced by FG4592 after OGD/R (Fig. 7A-D). Activation of UPR always leads to the expansion of the endoplasmic reticulum (ER) [50]. Thus, the innate ER protein calreticulin (CALR) was used to label the ER in the immunofluorescence images. As shown in Fig. 7E, F, compared with the vehicle group, the ER areas of primary cortical neurons in the FG4592 group exhibited significant expansion. Moreover, pretreatment with YC-1 did not hinder this expansion. Consistent with the in vitro results, the pretreatment of YC-1 did not inhibit the expression of sATF6, sXBP1 or p-eif2ɑ in peri-infarct brain tissue of FG4592-treated mice after 24 h of tMCAO (Fig. 7G, H). These data indicated that FG4592 could activate the UPR in a HIF-independent way, which may be mediated by the alternative pathway inhibiting OGFOD1.

**Fig. 8:**
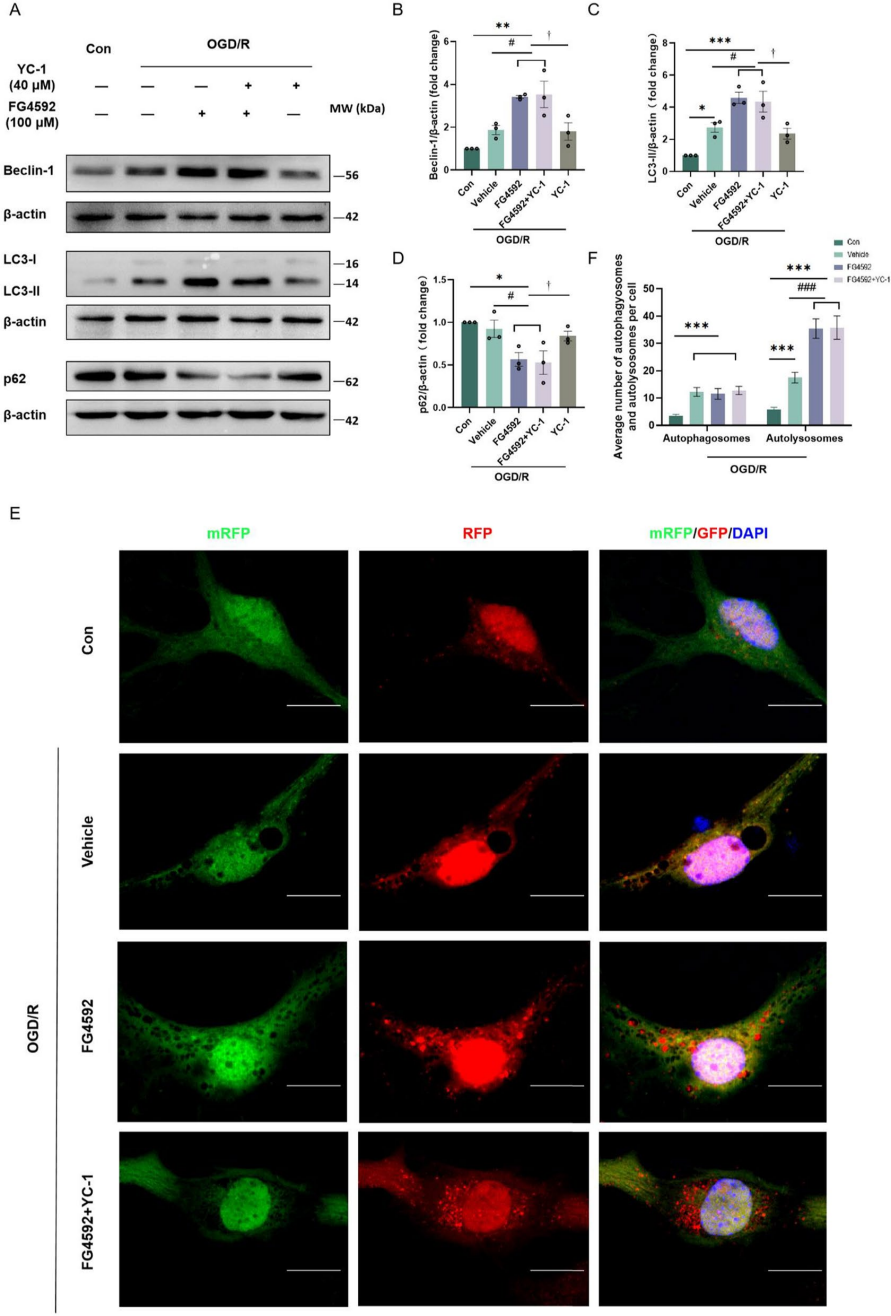
FG4592 activates autophagy flux in a HIF-1α independent way in primary cortical neurons. **A** Western blots analysis of autophagic proteins in the primary cortical neurons, including Beclin-1, LC3-II and p62/SQSTM1. Cells of YC-1 groups were first pretreated with YC-1 for 1 hour. All experimental groups were dealt with OGD for 3 hours and then treated with vehicle or FG4592 after reperfusion. **B-D** The bar graphs of western blot data in panel A. β-actin was used as a loading control. Data are presented as mean ± SEM, Con vs other groups: ***p < 0.001, **p < 0.01, *p < 0.05. vehicle vs FG4592 or FG4592+YC-1: #*p* < 0.05. YC-1 vs FG4592 or FG4592+YC-1: †*p* < 0.05 (one-way ANOVA followed by Dunnett’s *post-hoc *test, n = 3). **E** Representative confocal microscopy images correspond to the autophagic flux analysis for primary cortical neurons. Primary cortical neurons were transfected with mRFP-GFP-MAP1LC3B adenovirus for 4 days. Cells of the YC-1 group were pretreated with YC-1 for 1 hour. All experimental groups were dealt with OGD and then treated with vehicle or FG4592 after reperfusion. Scale bar = 10 μm, 100×. **F** The number of yellow (autophagosomes) and red (autolysosomes) puncta were counted. Data represented as mean ± SEM from three independent coverslips. Con vs other groups: ***p < 0.001, vehicle vs FG4592 or FG4592+YC-1: ###*p* < 0.001 (one-way ANOVA followed by Dunnett’s *post*-*hoc *test, n = 40 neurons in each group)

FG4592 upregulated the autophagy-related proteins in peri-infarct tissue after tMCAO. Similarly, the protein levels of Beclin-1 and LC3-II in primary cortical neurons were higher in the FG4592 group than in the vehicle group after OGD/R. Conversely, p62 decreased in the FG4592 group. However, the upregulation of Beclin-1 or LC3-II and downregulation of p62 were not altered by pretreatment with YC-1 (Fig. 8A–D). Immunofluorescent staining revealed that FG4592 increased the autolysosomes in primary cortical neurons after OGD/R. However, there was no significant difference in the number of autophagosomes between the FG4592 and vehicle groups. Consistent with the western blot data, pretreatment with YC-1 did not block the autophagic flux (Fig. 8E, F).

To further evaluate that FG4592 promoted autophagy via the activation of UPR. 4-phenylbutyrate (4-PBA) was used to inhibit the activation of UPR pathways, which has been verified in a previous study [18]. Herein, we found that the up-regulation of Beclin-1 and LC3-II or down-regulation of p62/SQSTM induced by FG4592 were rescued by the pretreatment of 4-PBA in the primary cortical neurons following I/R injury (Additional file 1: Fig. S10A–D). In general, FG4592 can activate the UPR-inducing autophagy pathway by inhibiting OGFOD1, which is mediated in a HIF-independent manner.

### Inhibiting UPR attenuates the neuroprotective function of FG4592 in vivo

**Fig. 9:**
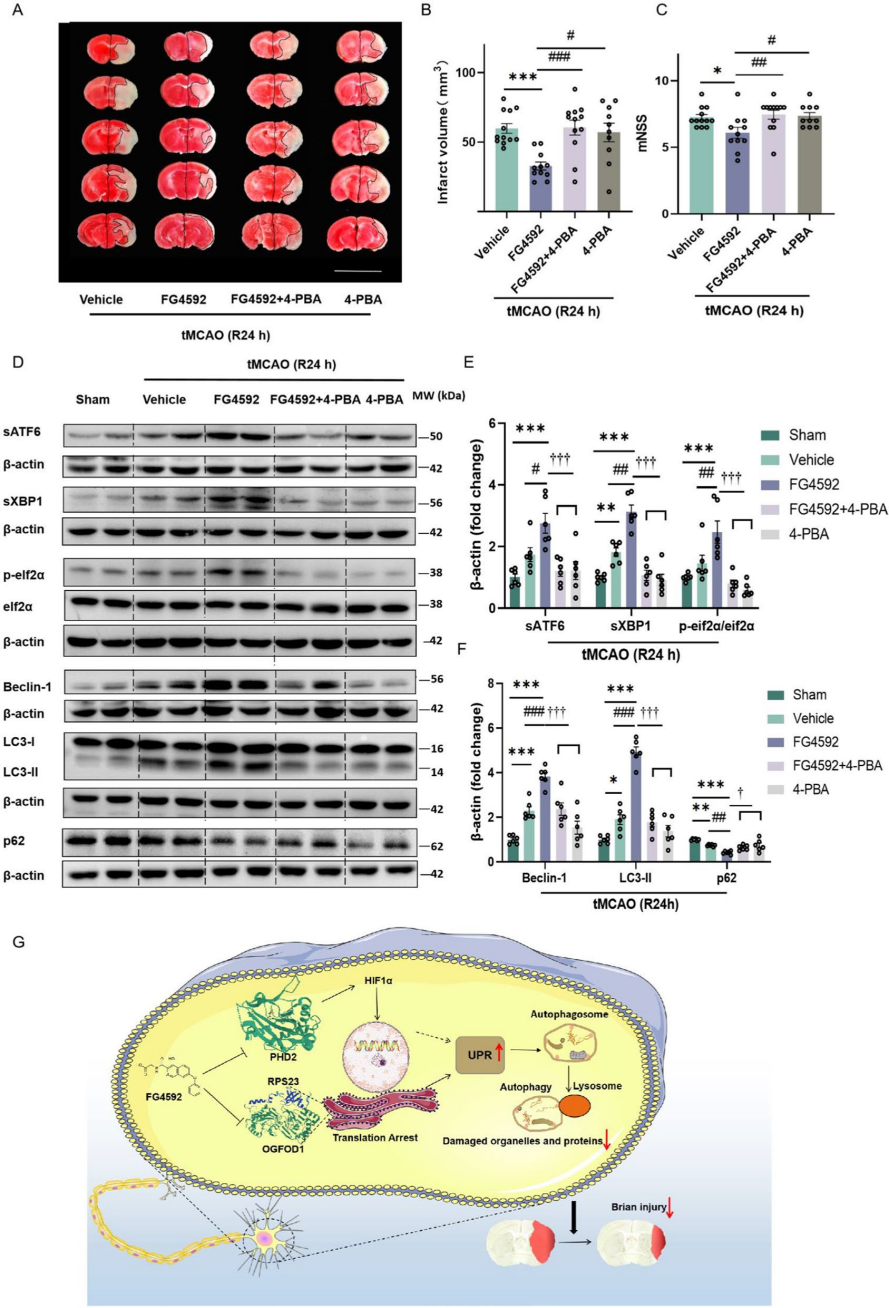
The neuroprotective function of FG4592 is attenuated by UPR inhibiting. **A **Representative TTC images corresponding to coronal brain sections from mice treated with vehicle (n = 12), FG4592 (5 mg/kg, n = 11), FG4592 + 4-PBA (inhibitor of ER stress, 42 mg/kg, n = 12) and 4-PBA (42 mg/kg, n = 10) on one day after tMCAO. 4-PBA was injected 1 hour before surgery. All experimental groups underwent 1 hour of tMCAO and 24 hours of reperfusion. Layer thickness = 1 mm, scar bar = 1 cm. **B **and **C** The infarct volumes (one-way ANOVA followed by Dunnett’s *post-hoc *test) and mNSS score (non-parametric Kruskal-Wallis test followed by Dunn’s *post-hoc*) were assessed 24 hours after reperfusion. Data are presented as mean ± SEM, vehicle vs FG4592: ***p < 0.001, *p < 0.01. FG4592 vs FG4592+4-PBA or 4-PBA: ###*p* < 0.001, ##*p *< 0.01, #*p *< 0.01. **D-F** Representative immunoblots and bar graphs of sATF6, sXBP1, p-eif2α, Beclin-1, LC3-II and p62 in peri-infarct tissue of mouse brains after 24 hours of tMCAO. The data are expressed as mean ± SEM. Sham vs vehicle or FG4592: ***p < 0.001, **p < 0.01, *p < 0.05. vehicle vs FG4592: ###*p* < 0.001, ##*p* < 0.01, #*p* < 0.05, FG4592 vs FG4592+4-PBA or 4-PBA: †††*p *< 0.001, †*p* < 0.05 (one-way ANOVA followed by the Dunnett’s *post*-*hoc *test. n = 6). **G** The schematic diagram for the activation role of FG4592 in UPR. The activation of UPR by FG4592 may involve two pathways. Namely, FG4592 can activate the UPR via inhabiting its novel target OGFOD1 or possibly inhibiting the classical target PHD2. The activation of UPR subsequently promotes autophagy and alleviates I/R injury

To verify whether the neuroprotective function of FG4592 is dependent on UPR activation in vivo. Mice were pretreated with three doses of 4-PBA (14, 42, or 140 mg/kg) and then subjected to tMCAO. According to sATF6 and sXBP1 expressions in peri-infarct mouse brain tissue, 4-PBA with a dose of 42 mg/kg was able to inhibit the expressions of sATF6 and sXBP1 in FG4592 treated mouse brain (Additional file 1: Fig. S11A-C). Based on the TTC-staining and mNSS score results, 4-PBA attenuated the neuroprotective function of FG4592 (Fig. 9A-C). As shown in Fig. 9D, E, 4-PBA significantly suppressed the expression of sATF6, sXBP1, and p-eif2α induced by FG4592 in peri-infarct brain tissue. Furthermore, up-regulation of Beclin-1, LC3-II and down-regulation of p62/SQSTM induced by FG4592 were rescued by 4-PBA in the mouse brain (Fig. 9D, F). The expression levels of Bcl2, Bax and cleaved-caspase-3 in the FG4592 group were also rescued by pretreatment with 4-PBA (Additional file 1: Fig. S12A, B). In summary, these in vivo data reflected that the neuroprotection of FG4592 was dependent on the activation of UPR, which further regulated the autophagy pathway after I/R injury.

## Discussion

The present study provides the first evidence that FG4592 can prevent ischemic brain injury and promote the recovery of neurological defects via the non-classical target of FG4592. First, FG4592 decreased infarct volumes and improved short-term and long-term neurological defects in mice after tMCAO or dMCAO. FG4592 also improved the histological outcome over an extended period, which suggested that the neuroprotective effect is not transient. An equal neuroprotective effect of FG4592 was also found in aged mice, female mice and mice with a preexisting comorbidity. Moreover, FG4592 activated autophagy and inhibited apoptosis in the brain after ischemia. Second, inhibiting the hypoxia-sensing pathway did not abolish the neuroprotective function of FG4592 in I/R models in vitro or in vivo. Third, FG4592 inhibited the hydroxylation of RPS23 by its potential target, OGFOD1. Inhibition of OGFOD1 activated the UPR and autophagic pathway, further alleviating ischemic injury in vitro and in vivo. Finally, FG4592 also activated the UPR and autophagic flux in a HIF-ɑ independent way. Inhibition of the UPR rescued the neuroprotective effect of FG4592. These findings demonstrated that inhibition of OGFOD1 by FG4592 plays a beneficial role in ischemic stroke by upregulating the UPR and UPR-inducing autophagy (Fig. 9G).

As a major healthcare burden in most countries, the prevention and treatment of ischemic stroke are high priorities. However, the limited time window of reperfusion treatments and lack of neuroprotective drugs lead most ischemic stroke patients into dilemma. Many new agents have shown neuroprotective effects in lab tests but failed during clinical trials [4, 51]. PHDs are the main enzymes regulating the expression of various genes and promoting cellular adaptation under hypoxia. Over the past two decades, numerous researchers have sought to determine the neuroprotective function of PHDs inhibitors in stroke models. To date, all data suggest that the pretreatment or post-treatment with PHDs inhibitions ameliorate the adverse outcomes of ischemic stroke in mice [8]. As an approved PHDs inhibitor, FG4592 may have protective effects in various diseases [10]. In line with these findings, we found that the FG4592 alleviated the I/R injury and improved short-term and long-term neurological recovery in mice subjected to permanent or transient ischemic stroke. More attention has been paid to the therapeutic time window of reperfusion therapies for decades. Previously, the time window was dependent on the time since stroke onset. However, more patients with salvageable tissue have been excluded from reperfusion therapies. Therefore, the tissue window assessed by perfusion imaging has been a consensus of clinicians, which helps to select patients with more extensive salvageable tissue for reperfusion therapies. Recently, the target window of different neuroprotection therapies would depend on different elements of the neurovascular unit at different times [12]. In the present study, FG4592 showed a significant reduction in infarct size of mice with 6- and 12-h delays after I/R injury but a slight reduction in the functional deficit. This delayed treatment window is a potential and supplementary therapy for patients who underwent reperfusion strategies.

Molecular analyses indicated that FG4592 upregulates the expression of HIF-1/2α and its target genes in peri-infarct tissue, which is consistent with the findings of preclinical studies using other PHDs inhibitors [8]. Moreover, FG4592 significantly activated the autophagy pathway and inhibited the apoptosis pathway in the brain tissue after I/R injury. Similarly, a recent study reported a pro-survival effect of FG4592 on BMSCs via autophagy activation and apoptosis inhibition and that transplantation of FG4592-preconditioned BMSCs promoted recovery from ischemic stroke in rats [11]. Most noticeably, the majority of anemia patients caused by chronic kidney disease are using FG4592. These anemia patients will be the optimal cohort for verifying the neuroprotection of FG4592.

In recent years, increasing studies have reported that protection via PHD inhibitors may not only depend on the upregulation of HIF-α, and most researchers have recently recognized this concept. For instance, some PHD inhibitors (DMOG) also inhibited inflammation or protected cells against oxidative death, even knock-down of cellular *Hif-1α* and *Hif-2α* [32, 34, 52]. After conditional knockout of HIF-1α in neurons or astrocytes, the neuroprotection of the PHDs inhibitor remained [33]. In the present study, the single or combined inhibition of HIF-1α and 2α did not eliminate the neuroprotection of FG4592. As the primary regulator of HIFα and the main type of PHDs in the cortex [38], the knock-down of PHD2 did not affect the neuroprotection via FG4592 in our study, which is consistent with previous studies using *Phd*2 + / − or neural-specific *Phd*2-deficient mice [53, 54]. Therefore, other potential targets or mechanisms of FG4592 warrant exploration.

Molecular docking is now widely used to develop drugs, and it can help reduce complicated steps and visualize spatial relationships between small molecules and proteins [26, 55]. With the help of Ledock software and online predicting methods, OGFOD1, FTO, and UTX/KDM6a were identified as potential targets of FG4592. OGFOD1 has a similar protein structure and active site as PHD2. Moreover, FG4592 inhibits the OGFOD1 with a lower IC50 value [39]. Meanwhile, this study demonstrated that FG4592 could inhibit the interaction between OGFOD1 and RPS23 and the hydroxylation of RPS23, which is in accord with the results of the *Ogfod*1 knockdown [46].

Inhibition or knockdown of *Ogfod1* is known to activate the UPR [44, 46]. UPR is an intrinsic mechanism to clear damaged or unfolded proteins and alleviate cell protein burden. The activation of UPR can restore endoplasmic reticulum function impaired by stress. It has been verified that knock-out p-eif2α, ATF6 and sXBP1 impair stroke outcomes in mice based on infarct volume and behavioral tests [48, 56–58]. On the contrary, knock-in or overexpression of *Xbp1s* or *sATF6* improves the outcomes of rodent animals after stroke or ischemia [57, 59, 60]. Moreover, activators, including 147 and Salubrinal, also showed neuroprotective function in mice that underwent tMCAO [48, 61]. In the present study, the activation of UPR was found in the HT-22 cells transfected with *Ogfod1* siRNA after the OGD/R. A recent study suggests that the knock-out of *Ogfod*1 significantly improves the outcomes of acute myocardial infarction and activates the UPR after the I/R injury [47]. Consequently, the inhibition of OGFOD1 may be a potential therapeutic target for ischemic stroke or other ischemic diseases, and FG4592 may exert its neuroprotective effects by inhibiting OGFOD1.

In the present study, the OGFOD1 was found to be a potential target of FG4592. FG4592 also activated the UPR pathway after I/R injury, which was not affected by the inhibition of HIF-1α by YC-1. The inhibition of OGFOD1 by FG4592 may promote the activation of UPR in an alternative way and mediate the neuroprotection of FG4592 in I/R injury. However, some studies also reported that the pretreatment of drugs to inhibit ER stress showed beneficial effects on stroke animal models [62]. In contrast, Zhang et al. found that the post-treatment of ER stress activators tunicamycin or thapsigargin significantly protected mice against I/R injury, which could be reserved by the autophagy inhibitor 3-MA[63]. UPR is closely related to ER stress and affects each other. Therefore, more researchers tended to regard that the appropriate activation of UPR can provide neuroprotection to the brain after the I/R injury.

UPR and autophagy can mutually activate and potentiate [30]. It is well established in the literature that appropriate activation of autophagy can protect the mouse brain against I/R injury [31]. In the present study, FG4592 activated the autophagic flux in a HIF-1α independent way. Moreover, the ER stress inhibitor (4-PBA) abolished the neuroprotection of FG4592 by inhibiting the activation of the UPR and autophagy pathways. These findings indicated that FG4592 inhibits the non-classical target OGFOD1, further activating the UPR and autophagy pathways and thus providing a neuroprotective effect through an alternative way. Nevertheless, the neuroprotection of autophagic activation is still controversial and may be a double-edged sword for acute ischemic stroke. The prolonged or excessive activation may harm the animals after stroke [30]. Therefore, the dose and course of drug treatment should be considered when regulating autophagy after ischemic stroke.

There are several limitations of this study. First, OGFOD1 is almost distributed in neurons even after the I/R injury. However, previous research reported that PHDs inhibitors could regulate the function of astrocytes or microglia in vitro or in vivo [64, 65]. Therefore, the neuroprotective effect of FG4592 in ischemic stroke can not only be attributed to the inhibition of OGFOD1. Second, the inhibition of OGFOD showed a neuroprotective effect after ischemia by activating the UPR and autophagy. However, the biological functions of OGFOD1 have not been recognized completely, and other hydroxylation substrates or undiscovered functions should be explored. *Ogfod*1 knock-out mice can be used to explore the mechanisms in further research. Third, previous studies indicated that the neuron-specific knock-out of *Phd*2 improved the recovery of mice after ischemic stroke [66]. However, the knockdown of P*hd2* in the present study did not impact the outcomes of mice after tMCAO, which might be attributed to the incomplete clearance of P*hd2*. Moreover, some prior studies also mentioned that the neuron-specific knock-out of *Phd*2 or *Phd2* + / − mice did not affect the recovery of neurological defects of mice after ischemia [53, 54]. Lastly, the drug with a large therapeutic time window would benefit more patients who missed the reperfusion therapies [12, 13]. In the present study, FG4592 exhibited substantial neuroprotection at 12 h on infarct volumes after stroke onset. However, multiple endpoints and behavioural strategies should be comprised in the following study, which helps demonstrate a sustained benefit and ensure sufficient clinical application value.

In conclusion, this study provides crucial evidence that the first approved PHDs inhibitor, FG4592, can prevent brain injury and improve the recovery of neurological defects in mice after transient or permanent ischemic stroke. Notably, the neuroprotection of FG4592 was mediated by the activation of UPR and autophagy in a HIF-1α independent manner, which may involve the target OGFOD1.

### Supplementary Information

**Additional file 1: Figure S1.** Experimental design and the representative laser speckle contrast imaging. **Figure S2.** FG4592 improves stroke outcomes of mice subjected to transient or distal MCAO. **Figure S3.** FG4592 alleviated the ischemic injury of aged mice, female mice and mice fed with a high-fat diet. **Figure S4.** YC-1 can inhibit the expression of HIF-1α induced by FG4592. **Figure S5.** FG4592 activates autophagy and inhibits the apoptotic pathway in a HIF-1α independent way. **Figure S6.** The knock-down efficiencies of different *Phd2*-siRNA were verified in vivo. **Figure S7.** The probable targets of FG4592 basing molecular docking and the intracephalic distribution of OGFOD1. **Figure S8.** Effects of FG4592 on OGD/R-induced neuron injury in primary cortical neurons of mice. **Figure S9.** The intracellular distribution of OGFOD1 and LC–MS/MS analyses of the hydroxylation in RPS23 immunoprecipitated by Anti-RPS23 from the lysate of HT-22 cell. **Figure S10.** 4-PBA inhibits the activation of autophagy induced by FG4592 in primary cortical neurons. **Figure S11.** The pretreatment of 4-PBA can inhibit the expression of sATF6 and sXBP1 induced by FG4592 after tMCAO. **Figure S12.** 4-PBA abolishes the anti-apoptosis function of FG4592 in tMCAO mouse brains.

**Additional file 2: Table S1.** Predicted targets of FG4592 by SEA search server. **Table S2.** Predicted targets of FG4592 by Swiss Target Prediction. **Table S3.** 2-oxoglutarate-dependent dioxygenases with definite structures. **Table S4.** The hydroxylation area of RPS23.

## Data Availability

All relevant data are available within the manuscript and the Supplementary Materials. Requests for more materials should be addressed to Zhijun Zhang (janemengzhang@vip.163.com).

## Abbreviations

ANOVA: One-way analysis of variance
BMSCs: Bone marrow stromal cells
CBF: Cerebral blood flow
CCK-8: Cell Counting Kit-8
dMCAO: Distal middle cerebral artery occlusion
EPO: Erythropoietin
ER: Endoplasmic reticulum
GLUT-1: Glucose transporter-1
HIF: Hypoxia- inducible factor
IC50: Half maximal inhibitory concentration
I/R: Ischemia–reperfusion
mNSS: Modified neurological severity score
OGD/R: Oxygen–glucose deprivation/reoxygenation
OGFOD1: 2-Oxoglutarate and iron-dependent oxygenase domain-containing protein
PHDs: Prolyl hydroxylases
Rps23: Small ribosomal protein s23
tMCAO: Transient middle cerebral artery occlusion
TTC: 2,3,5-Triphenyl Tetrazolium chloride
UPR: Unfolded protein response
VEGF: Endothelial growth factor
