# Supplementary material for: Inhibition of OGFOD1 by FG4592 confers neuroprotection by activating unfolded protein response and autophagy after ischemic stroke
Source: J Transl Med. 2024 Mar 7;22:248. doi: 10.1186/s12967-024-04993-3 (PMC10921652; doi:10.1186/s12967-024-04993-3)
Supplement: Supplementary file 1 — Additional file 1: Figure S1. Experimental design and the representative laser speckle contrast imaging. Figure S2. FG4592 improves stroke outcomes of mice subjected to transient or distal MCAO. Figure S3. FG4592 alleviated the ischemic injury of aged mice, female mice and mice fed with a high-fat diet. Figure S4. YC-1 can inhibit the expression of HIF-1α induced by FG4592. Figure S5. FG4592 activates autophagy and inhibits the apoptotic pathway in a HIF-1α independent way. Figure S6. The knock-down efficiencies of different Phd2-siRNA were verified in vivo. Figure S7. The probable targets of FG4592 basing molecular docking and the intracephalic distribution of OGFOD1. Figure S8. Effects of FG4592 on OGD/R-induced neuron injury in primary cortical neurons of mice. Figure S9. The intracellular distribution of OGFOD1 and LC–MS/MS analyses of the hydroxylation in RPS23 immunoprecipitated by Anti-RPS23 from the lysate of HT-22 cell. Figure S10. 4-PBA inhibits the activation of autophagy induced by FG4592 in primary cortical neurons. Figure S11. The pretreatment of 4-PBA can inhibit the expression of sATF6 and sXBP1 induced by FG4592 after tMCAO. Figure S12. 4-PBA abolishes the anti-apoptosis function of FG4592 in tMCAO mouse brains. [file 12967_2024_4993_MOESM1_ESM.docx]

**Supplementary data**

**Inhibition of OGFOD1 by FG4592 confers neuroprotection by activating unfolded protein response and autophagy after ischemic stroke**

**Jian Xie****^a^, Yuan Zhang****^b^, Bin Li^b^, Wen Xi^b^, Yu Wang^b^, Lu Li^b^, Chenchen Liu^b^, Ling Shen^b^, Bing Han^b^, Yan Kong^c^,** **HongHong Yao^b*^,** **Zhijun Zhang^a,c*^**

a Department of Neurology, Affiliated ZhongDa Hospital, School of Medicine, Institution of Neuropsychiatry, Key Laboratory of Developmental Genes and Human Disease, Southeast University, Nanjing, Jiangsu, 210009, China;

b Department of Pharmacology, School of Medicine, Southeast University, Nanjing, Jiangsu, 210009, China;

c Department of Biochemistry and Molecular biology, school of Medicine, Southeast University, No. 87 Dingjiaqiao Road, Nanjing, Jiangsu 210009, China;

d The Brain Cognition and Brain Disease institute of Shenzhen Institute of Advanced Technology, Chinese Academy of Sciences, Shenzhen, Guangdong,518055, China.

*Corresponding authors. E-mail addresses: [janemengzhang@vip.163.com](mailto:janemengzhang@vip.163.com) (Zhijun Zhang), and [yaohh@seu.edu.cn](mailto:yaohh@seu.edu.cn) (HongHong Yao).

**
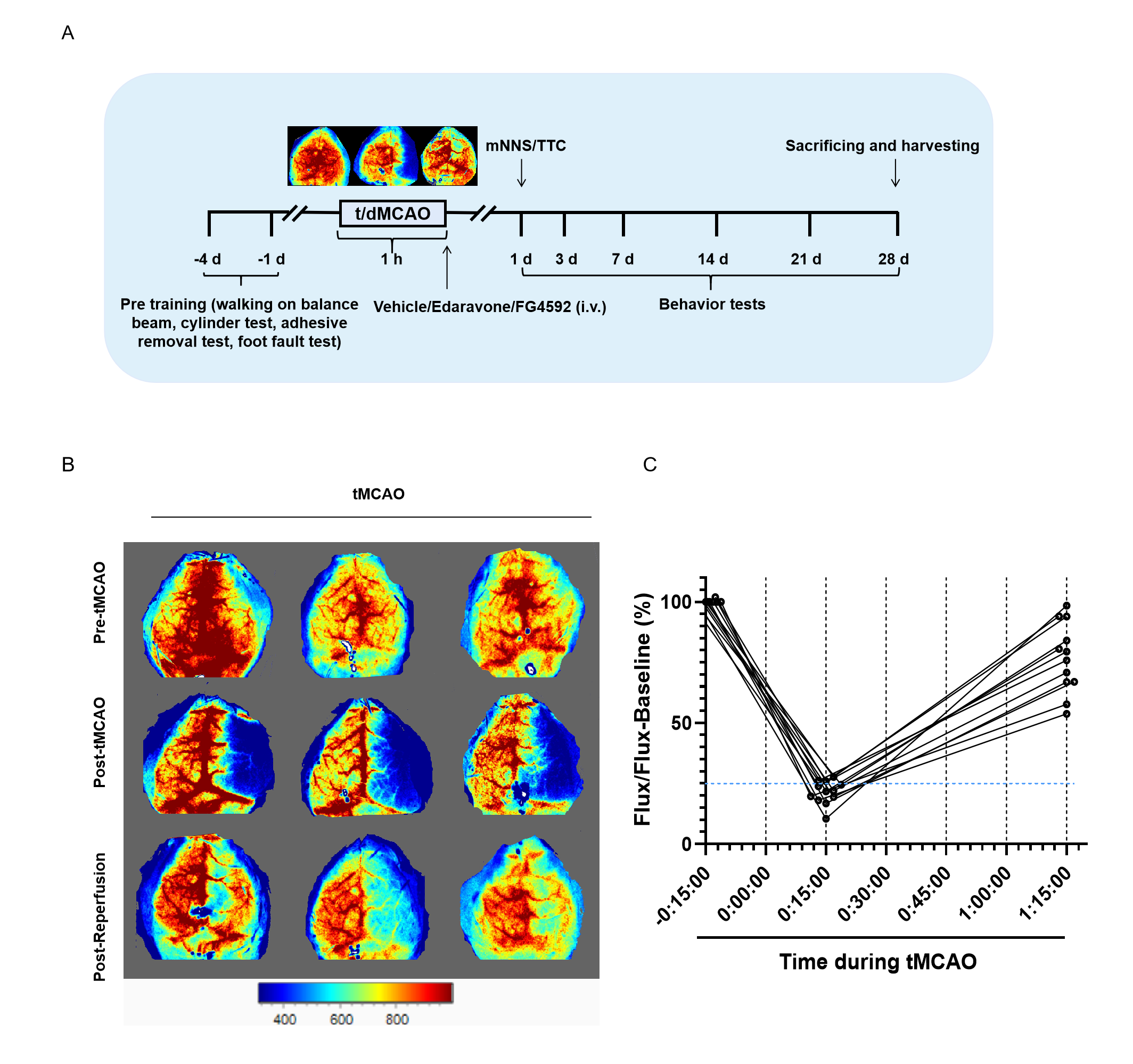
**

**Additional file 1: Fig. S1. Experimental design and the representative laser speckle contrast imaging. A** The timeline of behavioral pre-training, drug administration, TTC staining and behaviour testing. tMCAO was established on trainee mice. Vehicle and FG4592 (5 minutes after reperfusion, once every two days) and Edaravone (3 mg/kg, twice injection after 5- and 30-minutes following reperfusion or 1h following dMCAO, 6 mg/kg for the next 13 days, once a day) were administrated, followed by TTC staining and mNSS evaluation after 24 hours, and long-term behaviour tests. **B** The representative laser speckle contrast imaging of mice underwent tMCAO. **C** Mice were used in the following experiments based on the CBF of the ipsilateral and contralateral region of interest after tMCAO.

**
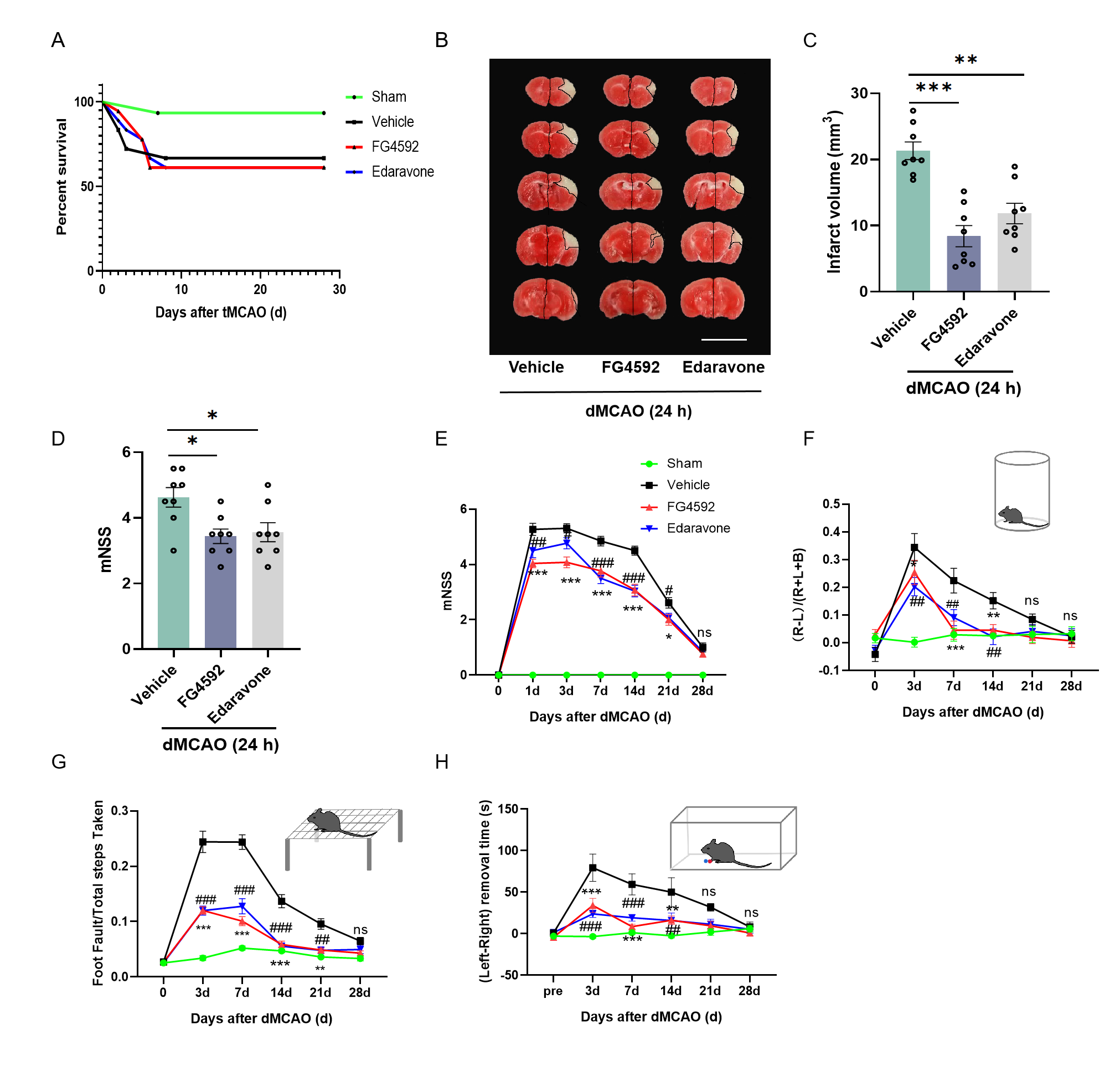
**

**Additional file 1: Fig. S2. FG4592 improves stroke outcomes of mice subjected to transient or permanent MCAO.** **A** The survival percentages of mice subjected to different treatments during the 28 days after tMCAO in mice. **B** The representative TTC staining of mouse brains after 24 hours of dMCAO. All mice were treated with vehicle, FG4592 (5 mg/kg) or Edaravone (3 mg/kg, twice after reperfusion). Layer thickness = 1 mm, scar bar = 1 cm. **C** and **D** Infarct volume (one-way ANOVA followed by Dunnett’s *post*-*hoc* test) and mNSS score (non-parametric Kruskal-Wallis test followed by Dunn’s *post-hoc*) of vehicle (n = 8), FG4592 (n = 8) and Edaravone (n = 8) groups after 24 hours of dMCAO. Data are presented as mean ± SEM. vehicle vs FG4592 or Edaravone: ****p* < 0.001, ***p* < 0.01, **p* < 0.05. **E**-**H** Sensorimotor functions were assessed by mNSS, cylinder test, foot fault task and adhesive removal test in the dMCAO mice till 28 days after surgery. Data are presented as mean ± SEM. FG4592 (n = 13) vs vehicle (n = 13): ****p* < 0.001, ***p* < 0.01, **p* < 0.05. Edaravone (n = 13) vs vehicle: ##*#p* < 0.001, #*#p* < 0.01, *#p* < 0.05 (Sham: n=12. There was not any death of mice after dMCAO. two-way repeated-measures ANOVA followed by Holm-Sidak *post-hoc* multiple-comparison tests).


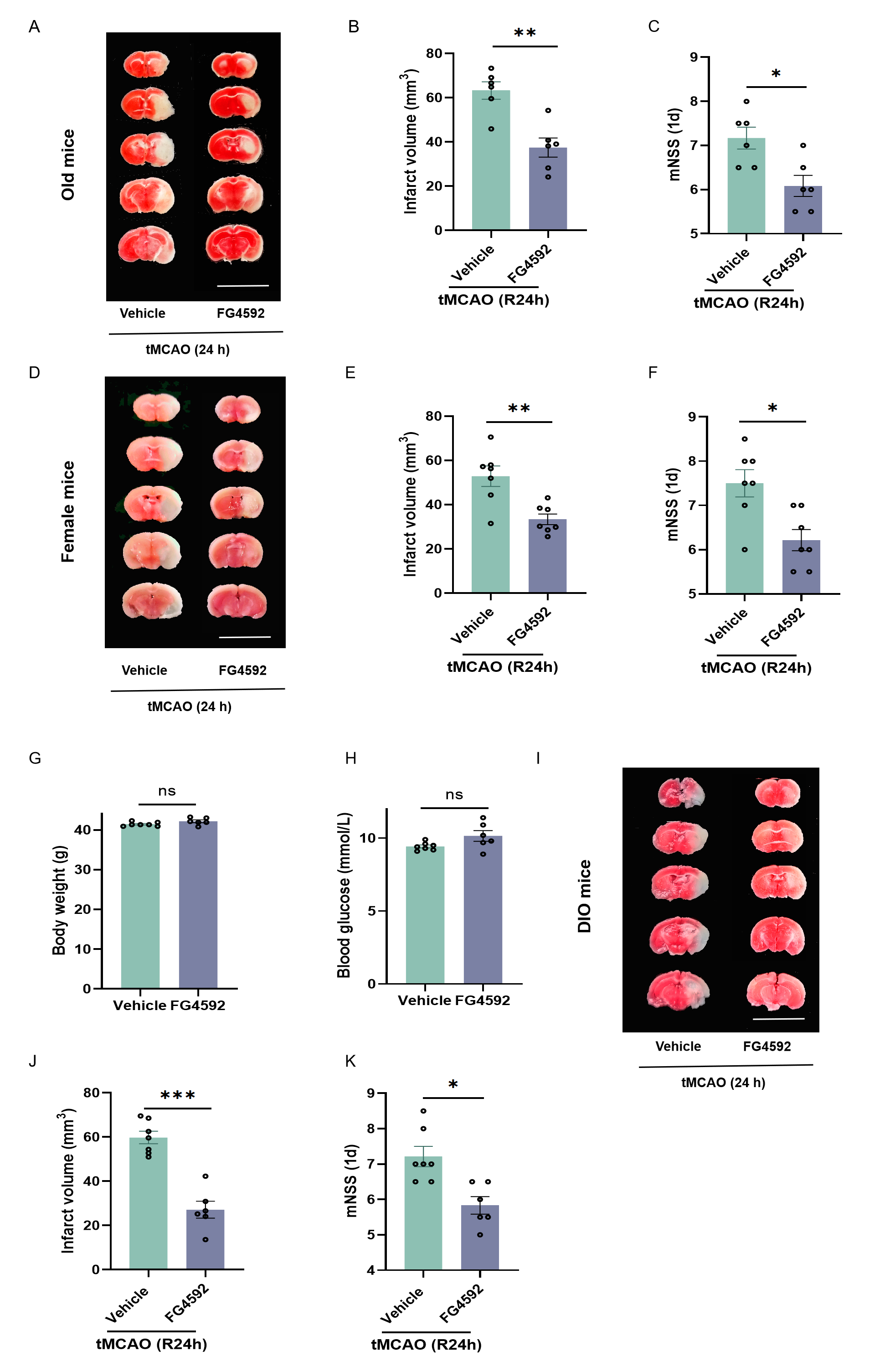


**Additional file 1: Fig. S3. FG4592 alleviated the ischemic injury of aged, female and mice fed with a high-fat diet.** **A** The representative TTC staining of aged mouse brains after 24 hours of tMCAO. All mice were treated with vehicle, FG4592 (5 mg/kg). Layer thickness = 1 mm, scar bar = 1 cm. **B** and **C** Infarct volume (unpaired Student’s *t*-test) and mNSS score (Mann–Whitney-U test) of vehicle (n = 7), FG4592 (n = 7) groups after 24 hours of tMCAO. **D** The representative TTC staining of female mouse brains after 24 hours of tMCAO. All mice were treated with vehicle or FG4592 (5 mg/kg). Layer thickness = 1 mm, scar bar = 1 cm. **E** and **F** Infarct volume (unpaired Student’s *t*-test) and mNSS score (Mann–Whitney-U test) of vehicle (n = 7), FG4592 (n = 7) groups after 24 hours of tMCAO. **G** and **H** The baseline body weight and blood glucose of DIO mice. **I** The representative TTC staining of DIO mouse brains after 24 hours of tMCAO. All mice were treated with vehicle or FG4592 (5 mg/kg). Layer thickness = 1 mm, scar bar = 1 cm. **J** and **K** Infarct volume (unpaired Student’s *t*-test) and mNSS score (Mann–Whitney-U test) of vehicle (n = 7), FG4592 (n = 7) groups after 24 hours of tMCAO.All data are presented as mean ± SEM. vehicle vs FG4592: ***p < 0.001, ***p* < 0.01, **p* < 0.05.


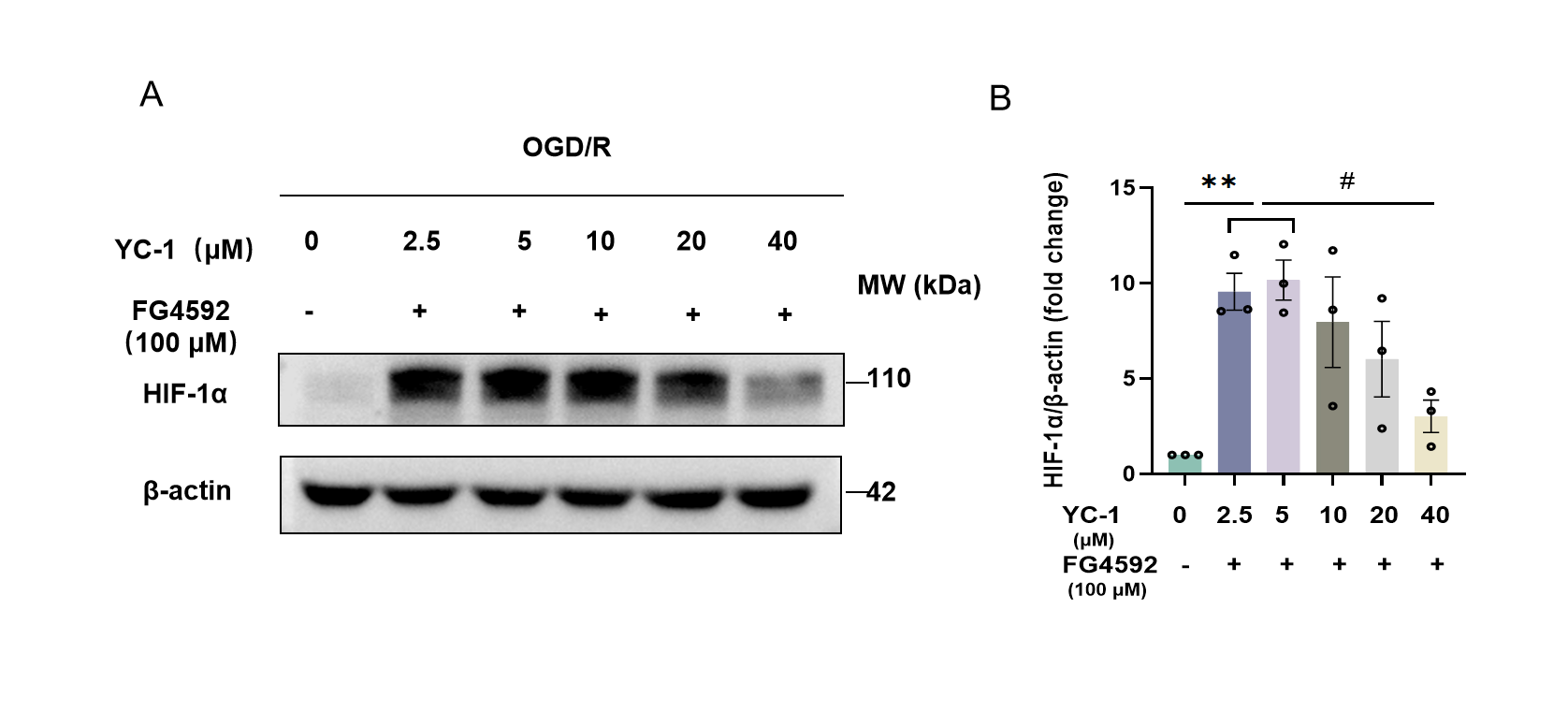


**Additional file 1: Fig. S4.** **YC-1 can inhibit the expression of HIF-1α induced by FG4592.** **A** and **B** Representative western blot and bar graph of HIF-1ɑ in HT-22 cells. Cells were pretreated with different concentrations of YC-1 (HIF-1ɑ inhibitor) 1 hour before OGD/R. All groups underwent OGD for 3 hours and were treated with vehicle or FG4592 (100 μM) 6 hours after reperfusion. FG4592+YC1 (0 μM) vs FG4592+YC-1 (2.5 μM) or FG4592+YC-1 (5 μM): ***p* < 0.01. FG4592+YC1 (40 μM) vs FG4592+YC-1 (2.5 μM) or FG4592+YC-1(5 μM）: *#p* < 0.05 (one-way ANOVA followed by Dunnett’s *post*-*hoc* test, n = 3).


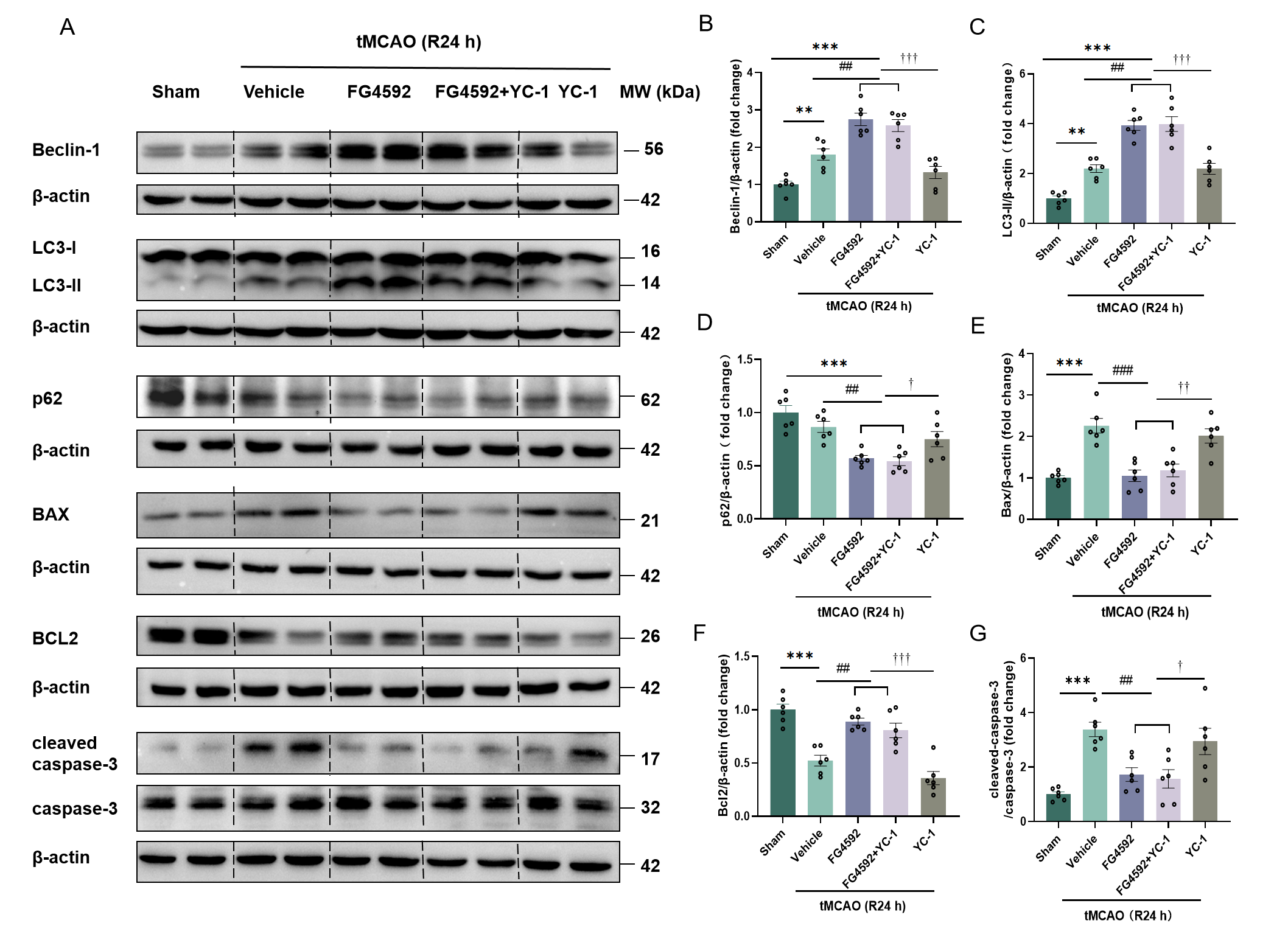


**Additional file 1: Fig. S5.** **FG4592 activates autophagy and inhibits the apoptotic pathway** **in a HIF-1α independent way. A** Images of western blots of Beclin-1, LC3-II, p62/SQSTM1, BAX, BCl2, cleaved-caspase-3 and caspase-3 in mouse brain after 24 hours of tMCAO. Mice pretreated with YC-1 were injected with YC-1 (intraperitoneally) 1 hour before tMCAO. **B**-**G** Western blot analyses of proteins are shown in panel A. Data are presented as mean ± SEM. Sham vs other groups: ****p* < 0.001, ***p* < 0.01. vehicle vs FG4592 or FG4592+YC-1 group: ##*#p* < 0.001, #*#p* < 0.01. YC-1 vs FG4592 or FG4592+YC-1 group: †††*p* < 0.001, ††*p* < 0.01, †*p* < 0.05 (one-way ANOVA followed by Dunnett’s *post*-*hoc* test, n = 6 in each group).


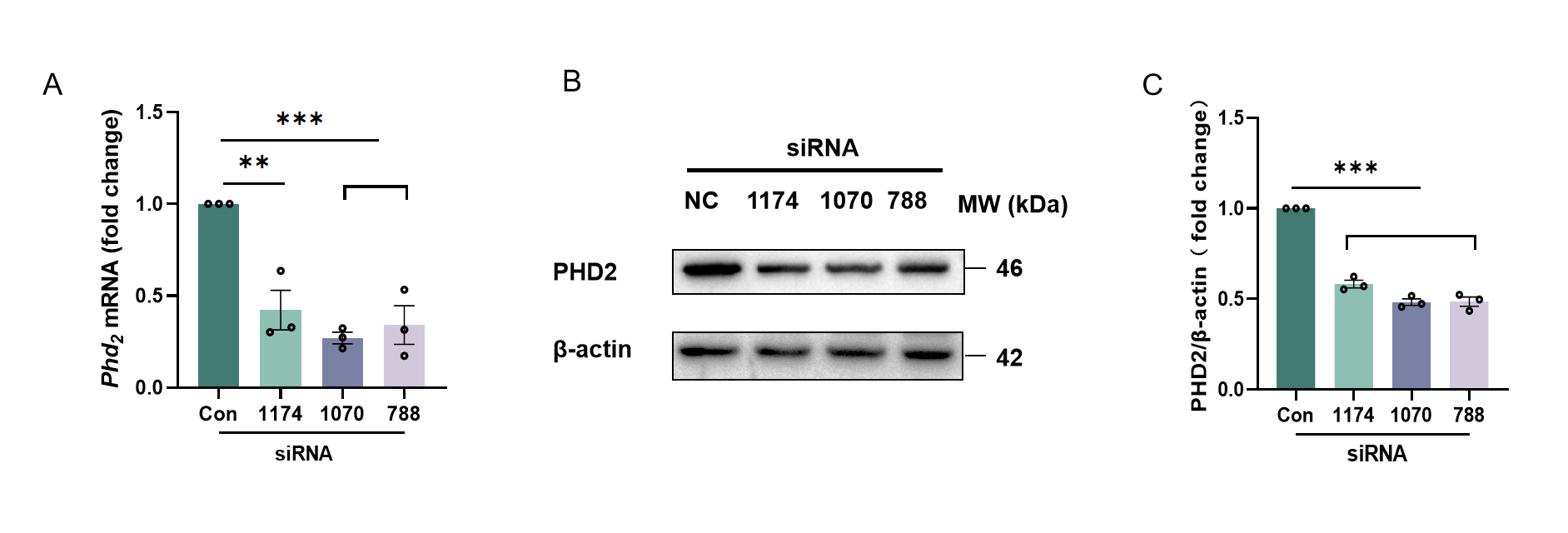


**Additional file 1: Fig. S6.** **The expression of PHD2 was downregulated with different *Phd2* siRNA**. **A** The mRNA levels of *Phd2* in HT-22 cells transfected with Con siRNA or *Phd2* siRNA for 36 hours. Data are presented as mean ± SEM. NC vs siRNA-1172 or siRNA-1070 or siRNA-788. ****p* < 0.001, ***p* < 0.01 (one-way ANOVA followed by Dunnett's *post*-*hoc* test, n = 3). **B** and **C** The representative western blot and protein level of PHD2 in HT-22 cells were transfected with Con siRNA or *Phd2* siRNA for 48 hours. Data are mean ± SEM. NC vs siRNA-1172 or siRNA-1070 or siRNA-788. ****p* < 0.001 (one-way ANOVA followed by Dunnett’s *post*-*hoc* test, n = 3).


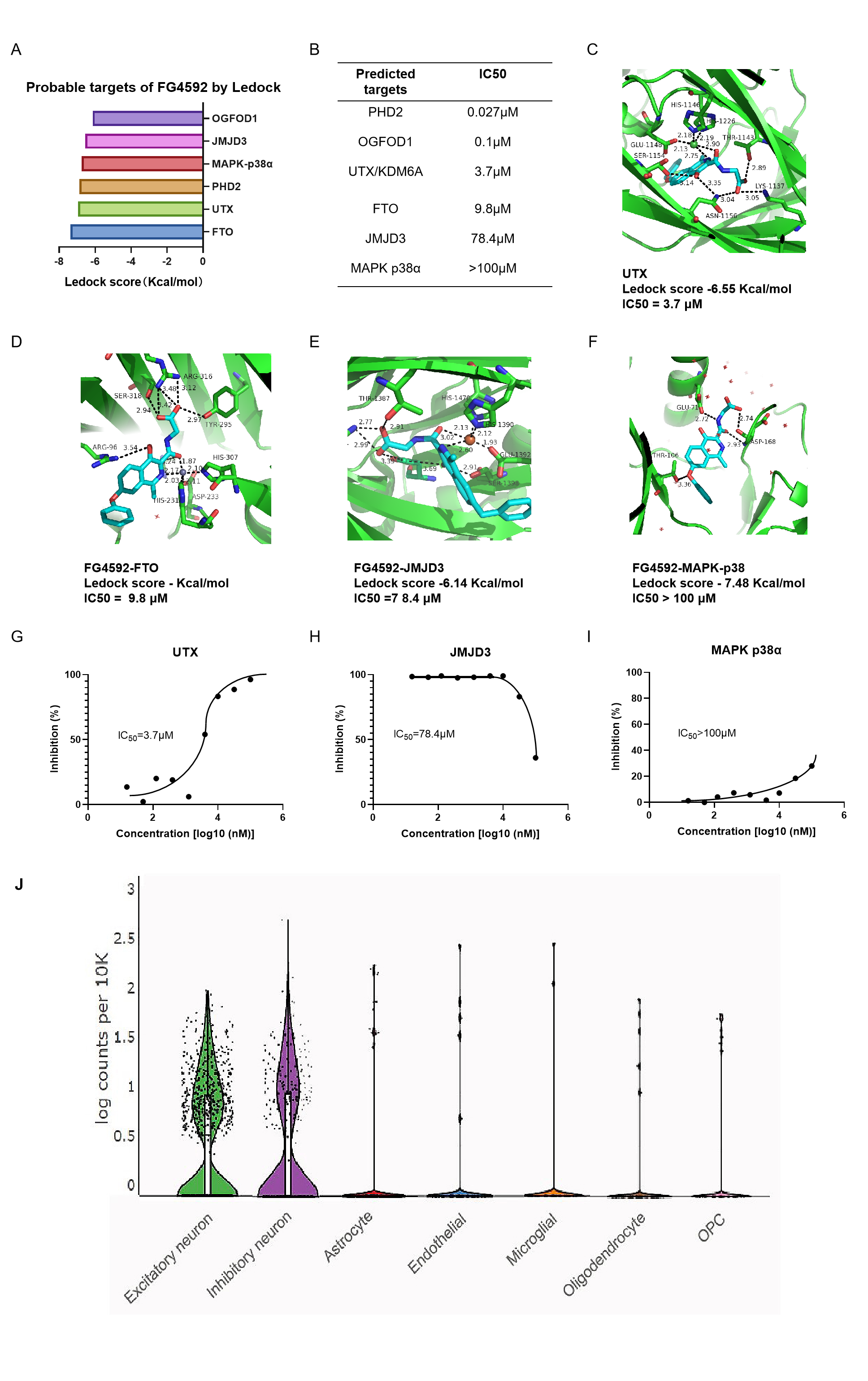


**Additional file 1: Fig. S7.** **The probable targets of FG4592. A** The Ledock score of FG4592 binding to different probable targets basing on online predicting and 2-oxoglutarate-dependent dioxygenases. **B** The 50 % inhibiting concentration (IC50) of probable targets was measured in vitro or reported in previous studies. **C**-**F** Docking analysis of FG4592 binding to UTX, FTO, JMJD3 and MAPK-p38. **G**-**I** In vitro analysis of the inhibition of GH4592 on UTX, JMJD3 and MAPK p38α. **J** The distribution of OGFOD1 in the central nervous system cell types according to the single cell portal database (Single cell port: Single Cell Comparison: Cortex data. Data provided by Joshua Levin et al.).

**
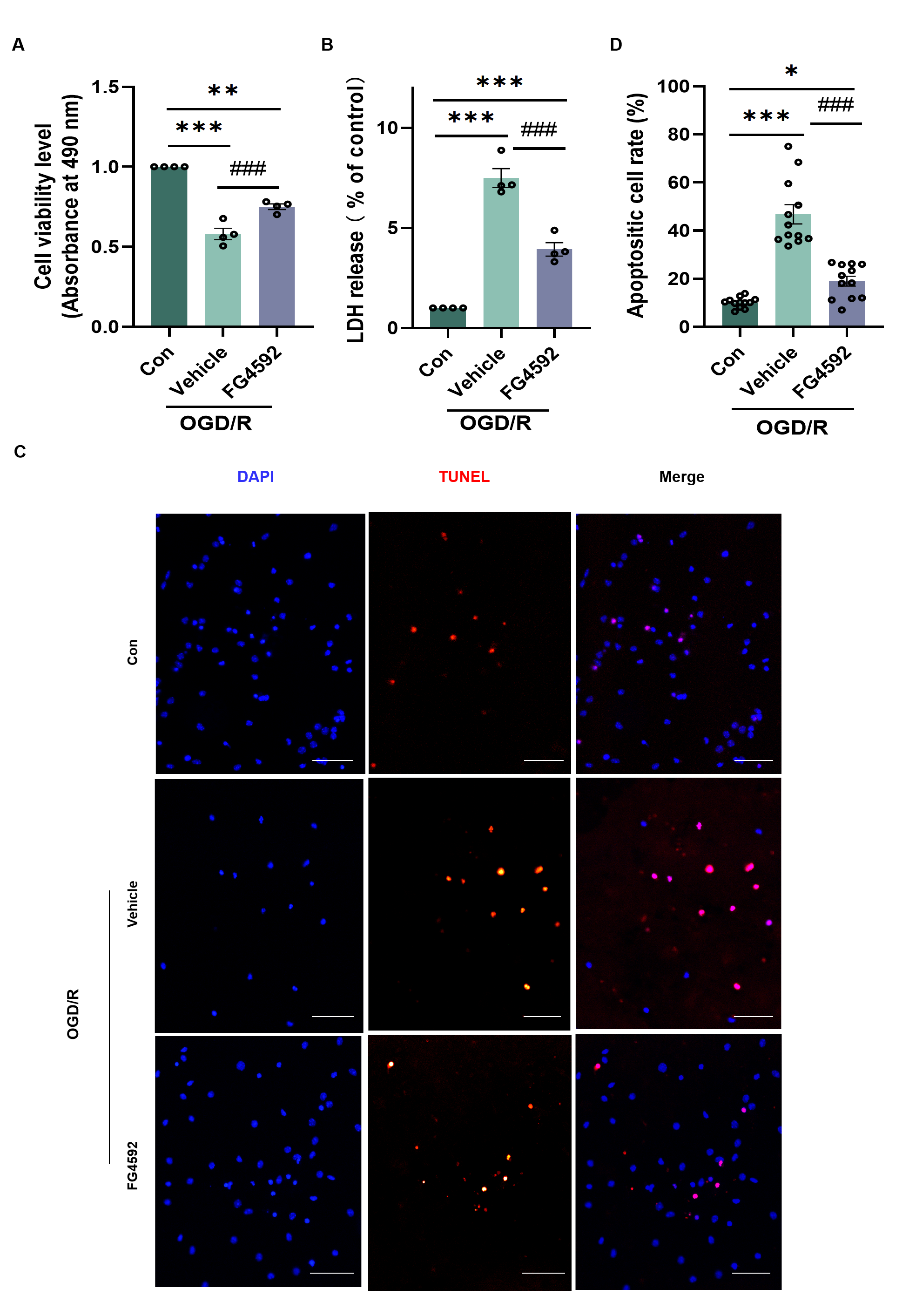
**

**Additional file 1: Fig. S8. Effects of FG4592 on OGD/R-induced neuron injury in primary cortical neurons of mice.** **A and B** The cell viability and LDH release assay of HT-22 cells was measured by the absorbance at 450 nm or 490 nm following OGD/R. Results are presented as mean ± SEM from four independent experiments. Con vs Vehicle or FG4592: ****p* < 0.001, ***p* < 0.01. Vehicle vs FG4592: ###*p* < 0.001 (one-way ANOVA followed by Dunnett’s *post*-*hoc* test). **C** Representative fluorescence micrographs of primary cortical neurons labeled with TUNEL staining. All experimental groups were dealt with OGD and then treated with vehicle or FG4592 after reperfusion. Scale bar = 50 μm. 20×. **D** The resulting histogram showing the percentage of apoptotic cells (featured by bright red signals) in the cell population after different treatments as indicated. Bars represent mean ± SEM (n = 12 fields from three independent coverslips). Con vs Vehicle or FG4592: ****p* < 0.001, **p* < 0.05. Vehicle vs FG4592: ###*p* < 0.001 (one-way ANOVA followed by Dunnett’s *post*-*hoc* test).


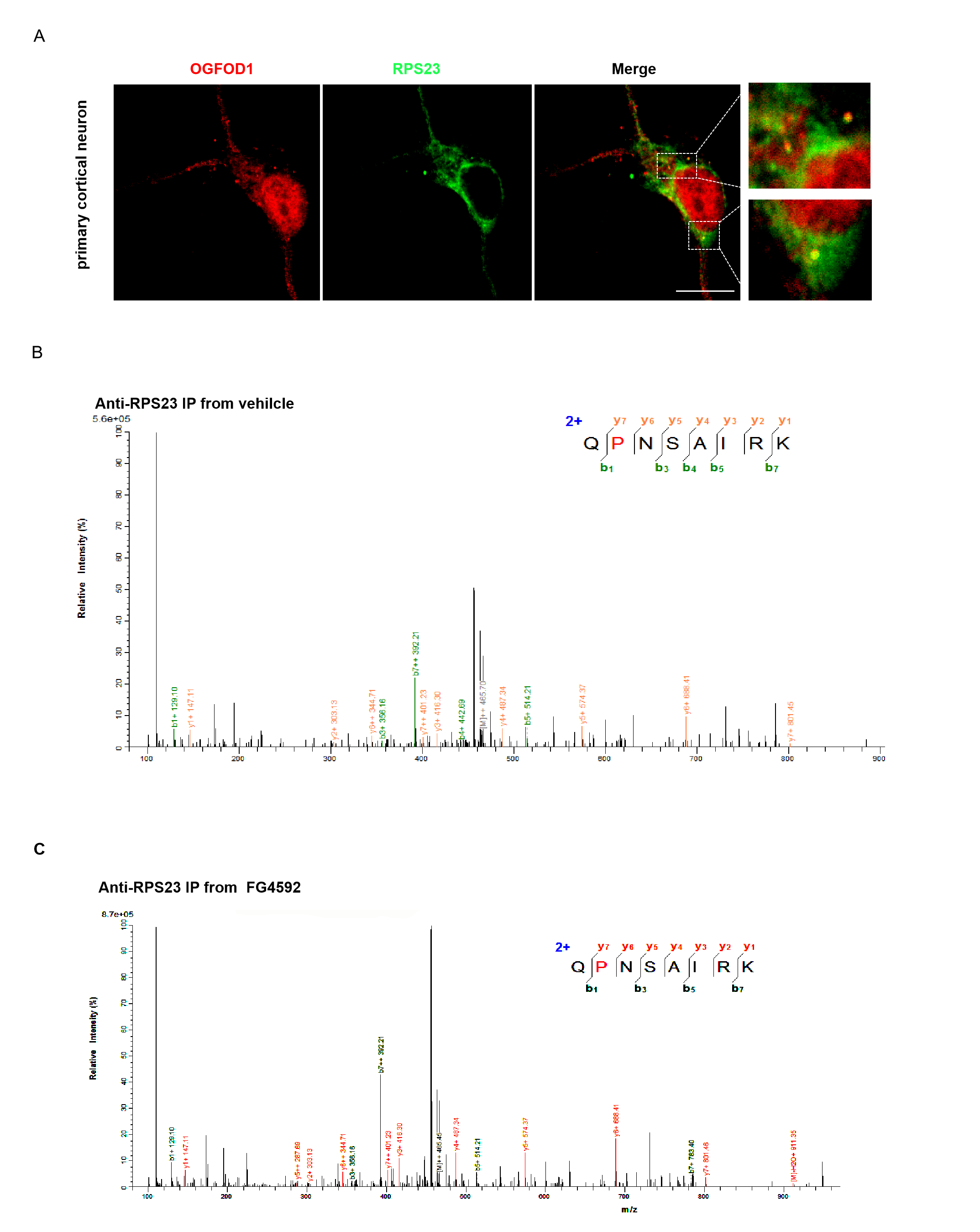


**Additional file 1: Fig. S9****. The intracellular distribution of OGFOD1 and**.**LC-MS/MS analyses of the hydroxylation in RPS23 protein. A** The immunofluorescence images of OGFOD1 and RPS23 in the primary cortical mice neuron which was cultured for 14 days in vitro (red, OGFOD1; green, RPS23). Scale bar, 10 μm, 100 ×. **B** and **C** RPS23 proteins were immunoprecipitated by Anti-RPS23 from the lysate of HT-22 cell pretreated with vehicle (B) or FG4592 (100 μM) (C) for 24 hours.


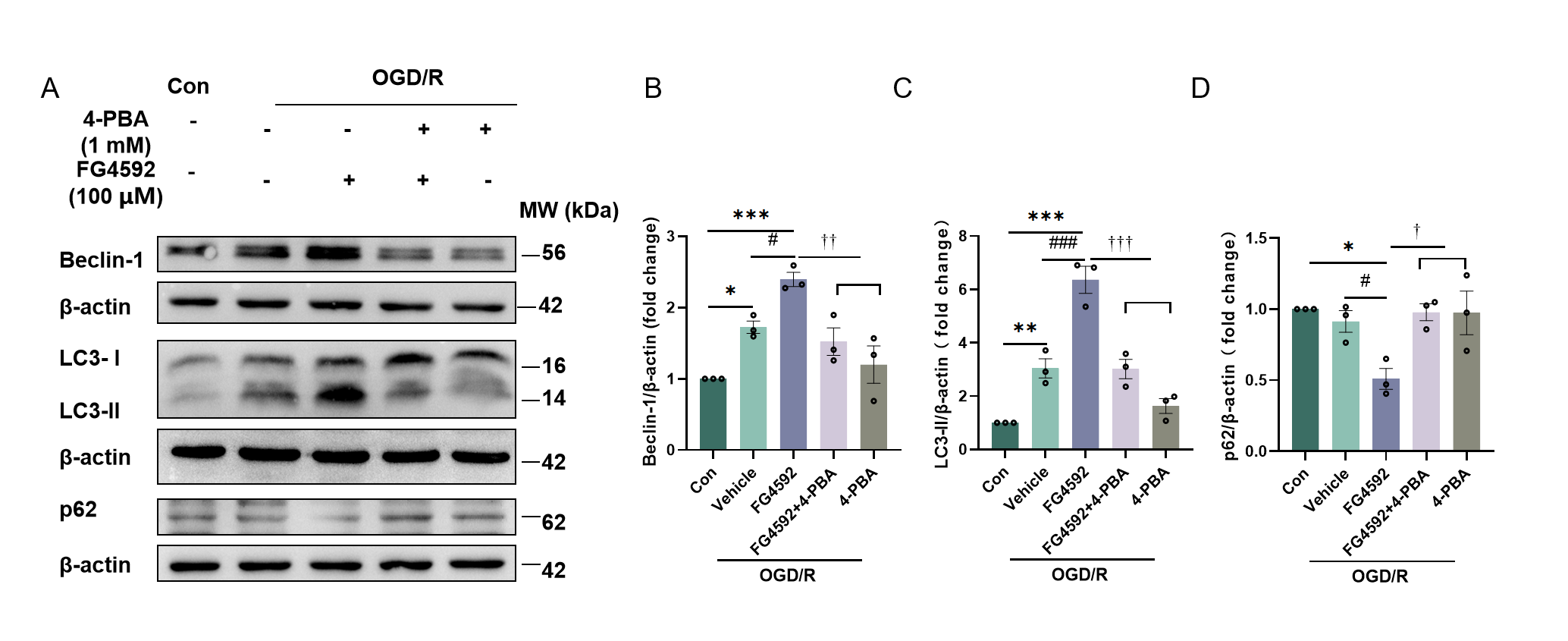


**Additional file 1: Fig. S10. 4-PBA inhibits the activation of autophagy induced by FG4592 in primary cortical neurons. A** The representative western blot images of Beclin-1, LC3-II, p62/SQSTM1 in primary cortical neurons after OGD/R. Cells were pretreated with 4-PBA (1mM) 1 hour before OGD/R and further treated with vehicle or FG4592 for 6 hours following OGD/R. **B**-**D** The statistical analysis of Beclin-1, LC3-II, p62/SQSTM1 in A panel. Data are presented as mean ± SEM. Con vs other groups: ****p* < 0.001, ***p* < 0.01, **p* < 0.05. vehicle vs FG4592: ###*p* < 0.001, #*p* < 0.05. FG4592 vs FG4592+4-PBA or 4-PBA: †††*p* < 0.001, ††*p* < 0.01, †*p* < 0.05 (one-way ANOVA followed by Dunnett’s post-hoc test, n = 3 in each group).


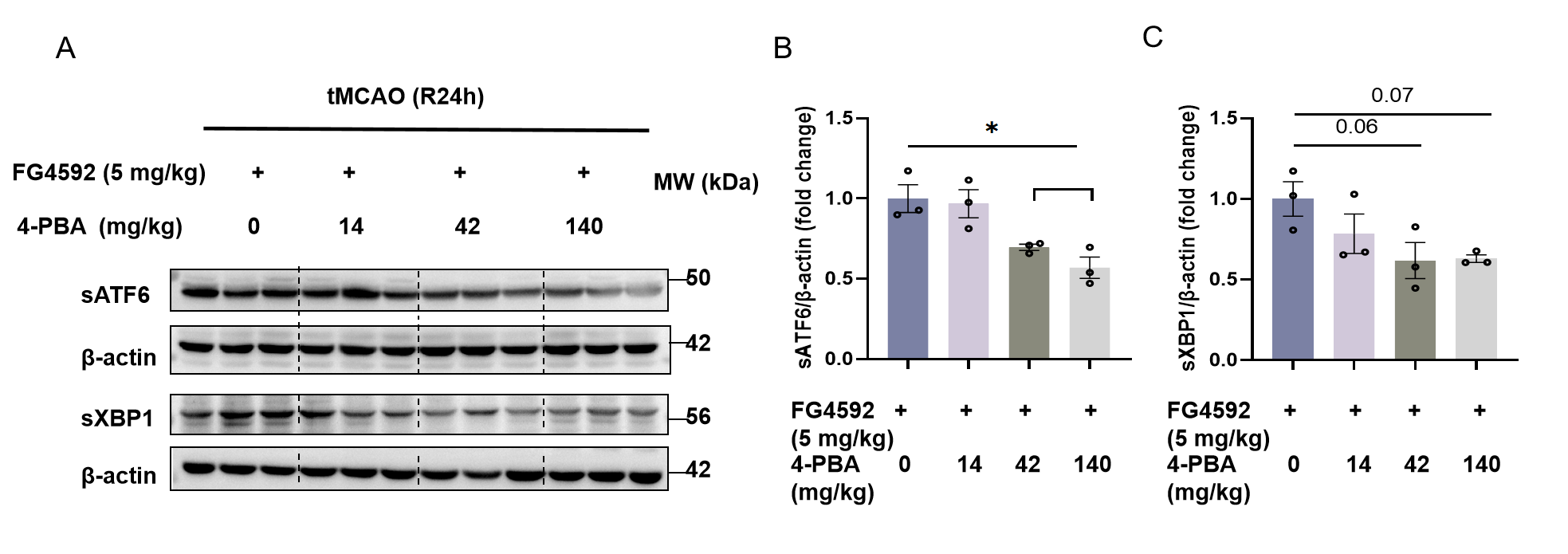


**Additional file 1: Fig. S11. The pretreatment of 4-PBA can inhibit the expression of sATF6 and sXBP1 induced by FG4592** **after tMCAO.** **A** The representative western blot of sATF6, sXBP1 in peri-infarct brain tissue of mice after 24 hours of tMCAO. Mice pretreated with 4-PBA were first injected with different doses of 4-PBA intraperitoneally before 1 hour of tMCAO. All mice were subjected to tMCAO and injected with FG4592 after tMCAO. **B** and **C** The statistical analysis of sATF6, sXBP1 A panel. Data are presented as mean ± SEM. FG4592 vs other groups: **p* < 0.05 (one-way ANOVA followed by Dunnett’s *post*-*hoc* test, n = 3 in each group).


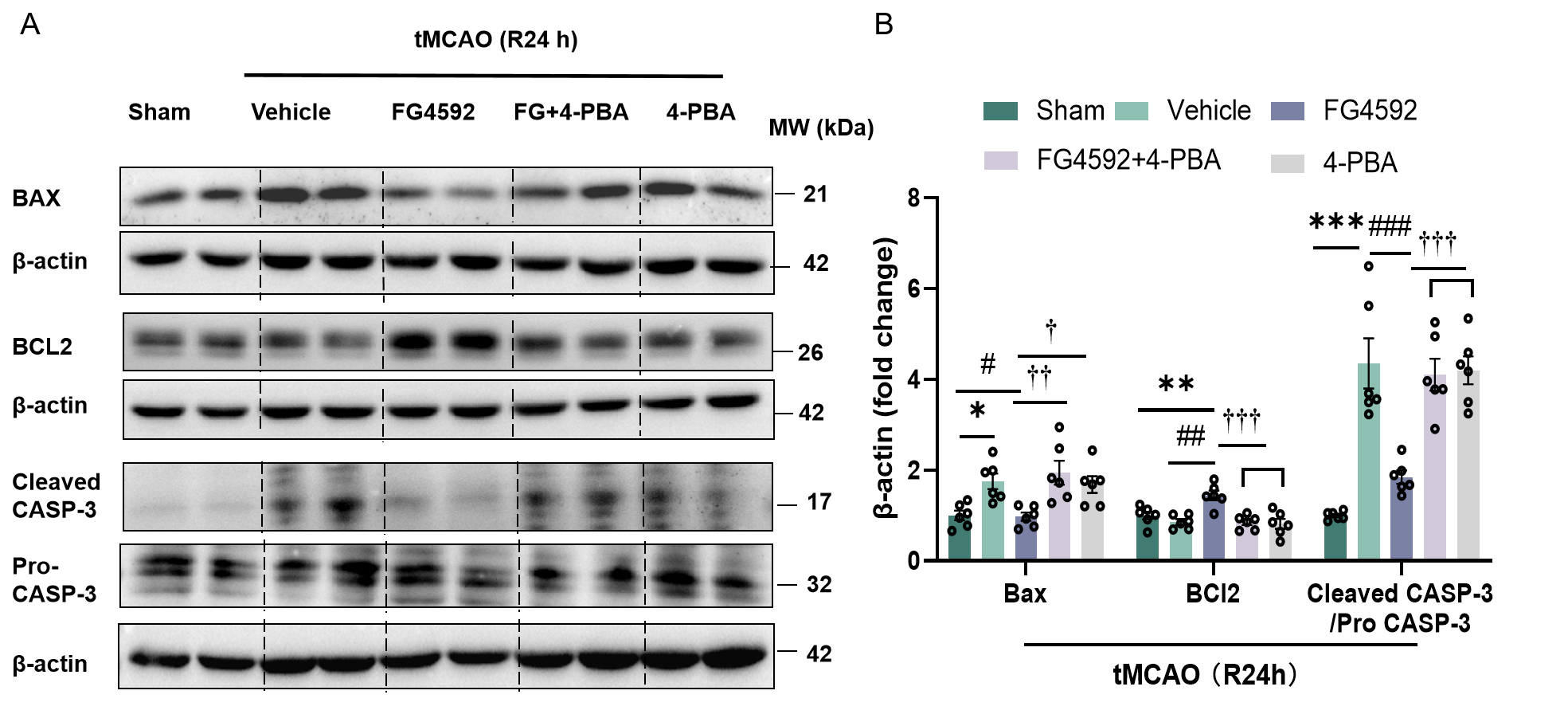


**Additional file 1: Fig. S12.** **4-PBA abolishes the anti-apoptosis function of FG4592 in mice brains after tMCAO. A** The representative western blots of BAX, BCL2, cleaved-caspase-3 and caspase-3 in peri-infarct tissue of tMCAO mouse brains. Mice were pretreated with 4-PBA (42 mg/kg, *ip*) 1 hour before tMCAO and treated with Vehicle or FG4592 (5 mg/kg, *i.v.*) after stroke. **B** The statistical analysis of proteins in panel A. Data are presented as mean ± SEM. Sham vs vehicle or FG4592: ****p* < 0.001, ***p* < 0.01, **p* < 0.05. vehicle vs FG4592: ###*p* < 0.001, ##*p* < 0.01, #*p* < 0.05. FG4592 vs FG4592+4-PBA or 4-PBA: †††*p* < 0.001, ††*p* < 0.01, †*p* < 0.05 (one-way ANOVA followed by the Dunnett’s *post*-*hoc* test. n = 6).
