# Supplementary material for: Inhibition of OGFOD1 by FG4592 confers neuroprotection by activating unfolded protein response and autophagy after ischemic stroke
Source: J Transl Med. 2024 Mar 7;22:248. doi: 10.1186/s12967-024-04993-3 (PMC10921652; doi:10.1186/s12967-024-04993-3)
Supplement: Supplementary file 2 — Additional file 2: Table S1. Predicted targets of FG4592 by SEA search server. Table S2. Predicted targets of FG4592 by Swiss Target Prediction. Table S3. 2-oxoglutarate-dependent dioxygenases with definite structures. Table S4. The hydroxylation area of RPS23. [file 12967_2024_4993_MOESM2_ESM.docx]

**Supplementary data**

**Inhibition of OGFOD1 by FG4592 confers neuroprotection by activating unfolded protein response and autophagy after ischemic stroke**

**Jian Xie****^a^, Yuan Zhang****^b^, Bin Li^b^, Wen Xi^b^, Yu Wang^b^, Lu Li^b^, Chenchen Liu^b^, Ling Shen^b^, Bing Han^b^, Yan Kong^c^,** **HongHong Yao^b*^,** **Zhijun Zhang^a,c*^**

a Department of Neurology, Affiliated ZhongDa Hospital, School of Medicine, Institution of Neuropsychiatry, Key Laboratory of Developmental Genes and Human Disease, Southeast University, Nanjing, Jiangsu, 210009, China;

b Department of Pharmacology, School of Medicine, Southeast University, Nanjing, Jiangsu, 210009, China;

c Department of Biochemistry and Molecular biology, school of Medicine, Southeast University, No. 87 Dingjiaqiao Road, Nanjing, Jiangsu 210009, China;

d The Brain Cognition and Brain Disease institute of Shenzhen Institute of Advanced Technology, Chinese Academy of Sciences, Shenzhen, Guangdong,518055, China.

*Corresponding authors. E-mail addresses: [janemengzhang@vip.163.com](mailto:janemengzhang@vip.163.com) (Zhijun Zhang), and [yaohh@seu.edu.cn](mailto:yaohh@seu.edu.cn) (HongHong Yao).

**Additional file 2: Table S1 Predicted targets of FG4592 by SEA search server**

| **Target Name** | **Description** | **P-Value** | **MaxTC** |
| --- | --- | --- | --- |
| EGLN1 | Egl nine homolog 1 | 1.00E-171 | 1 |
| FTO | Alpha-ketoglutarate-dependent dioxygenase FTO | 2.31E-57 | 1 |
| EGLN2 | Egl nine homolog 2 | 3.75E-235 | 0.6 |

**Additional file 2: Table S2 Predicted targets of FG4592 by Swiss Target Prediction**

| **Target** | **Common name** | **probability** | **Known actives (3D/2D)** |
| --- | --- | --- | --- |
| EGLN1 | Egl nine homolog 1 | 1 | 55/133 |
| FTO | Alpha-ketoglutarate-dependent dioxygenase FTO | 1 | 1/2 |
| EGLN3 | Egl nine homolog 3 | 0.11 | 3/8 |
| MAPK p38α | MAP Kinase p38 | 0.11 | 37/17 |

**Additional file 2: Table S3 2-OG dependent dioxygenases with definite structures**

| **Target** | **Common name** | **Ledock score** |
| --- | --- | --- |
| JMJD3 | Jumonji domain containing 3 | -6.14 |
| FTO | Alpha-ketoglutarate-dependent dioxygenase FTO | -6.75 |
| UTX | ubiquitously transcribed tetratricopeptide repeat, X chromosome | -6.55 |
| UTY | ubiquitously transcribed tetratricopeptide repeat,Y chromosome | -5.98 |
| ABH1 | alkylated DNA repair protein alkB homolog ABH1 | -4.20 |
| ASPH | aspartyl/asparaginyl b-hydroxylase | -5.93 |
| JMJD2D | Jumonji domain containing 2D | -6.22 |
| JMJD2E | Jumonji domain containing 2E | -6.21 |
| KIAA1718 | KIAA1718 | -4.77 |
| PHF8 | PHD finger protein 8 | -5.61 |
| PHYHD1 | PHYHD1 | -5.77 |
| OGFOD1 | 2OG, Fe dependent oxygenase domain 1 | -6.94 |
| BBOX | g-butyrobetaine hydroxylase | -6.88 |

**Additional file 1: Table S4 The hydroxylation area of RPS23**

| **Description** | **Amino acid** | **Oxidation (P) Probabilities** | **Con Area** | **FG Area** |
| --- | --- | --- | --- | --- |
| 40S ribosomal protein S23 | P | QP(1)NSAIRK | 10236000 | 4097000 |
